# Resistance to Hemi-Biotrophic *F. graminearum* Infection Is Associated with Coordinated and Ordered Expression of Diverse Defense Signaling Pathways

**DOI:** 10.1371/journal.pone.0019008

**Published:** 2011-04-20

**Authors:** Lina Ding, Haibin Xu, Hongying Yi, Liming Yang, Zhongxin Kong, Lixia Zhang, Shulin Xue, Haiyan Jia, Zhengqiang Ma

**Affiliations:** The Applied Plant Genomics Lab, National Key Lab of Crop Genetics and Germplasm Enhancement and Crop Genomics and Bioinformatics Center, Nanjing Agricultural University, Nanjing, Jiangsu, China; Instituto de Biología Molecular y Celular de Plantas, Spain

## Abstract

*Fusarium* species cause serious diseases in cereal staple food crops such as wheat and maize. Currently, the mechanisms underlying resistance to *Fusarium-*caused diseases are still largely unknown. In the present study, we employed a combined proteomic and transcriptomic approach to investigate wheat genes responding to *F. graminearum* infection that causes Fusarium head blight (FHB). We found a total of 163 genes and 37 proteins that were induced by infection. These genes and proteins were associated with signaling pathways mediated by salicylic acid (SA), jasmonic acid (JA), ethylene (ET), calcium ions, phosphatidic acid (PA), as well as with reactive oxygen species (ROS) production and scavenging, antimicrobial compound synthesis, detoxification, and cell wall fortification. We compared the time-course expression profiles between FHB-resistant Wangshuibai plants and susceptible Meh0106 mutant plants of a selected set of genes that are critical to the plants' resistance and defense reactions. A biphasic phenomenon was observed during the first 24 h after inoculation (hai) in the resistant plants. The SA and Ca^2+^ signaling pathways were activated within 6 hai followed by the JA mediated defense signaling activated around 12 hai. ET signaling was activated between these two phases. Genes for PA and ROS synthesis were induced during the SA and JA phases, respectively. The delayed activation of the SA defense pathway in the mutant was associated with its susceptibility. After *F. graminearum* infection, the endogenous contents of SA and JA in Wangshuibai and the mutant changed in a manner similar to the investigated genes corresponding to the individual pathways. A few genes for resistance-related cell modification and phytoalexin production were also identified. This study provided important clues for designing strategies to curb diseases caused by *Fusarium*.

## Introduction

Plants have evolved multiple layers of passive and active defense mechanisms to combat microbial pathogen attack in order to maintain their growth or survival. Passive defense takes advantage of preexisting structures [1] and preformed antimicrobial or toxic secondary metabolites, proteins, or peptides [2]. Active defenses, such as oxidative burst induction [3], hypersensitive response (HR) [4], accumulation of toxic compounds [5], and fortification of cell walls [6], are triggered rapidly and directly in response to pathogen attack. The timing of defense reaction activation and the strength of the defense response determine the resistance level.

Active plant defense is finely regulated to survive adversity at a minimum expense to growth. This regulation is multifaceted and might vary depending on the plant taxa and the pathogen lifestyle. The innate plant immunity system known as pathogen-associated molecular patterns (PAMP), or PAMP-triggered immunity (PTI) contributes to the first line of active defense through the Ca^2+^ signaling pathway, MAPK cascade, and transcriptome reprogramming that activate appropriate defense responses [7]–[10]. When PTI becomes ineffective because of pathogen effector generation, the second line of active defense, referred to as effector-triggered immunity (ETI), is required for resistance meditated by the interactions of host resistance (R) genes and pathogen effectors [7]. ETI, currently exclusively found in resistance against biotrophic pathogens that derive nutrients from living host tissues, is generally race-specific and results in HR at the infection site. Concomitant with the accumulation of salicylic acid (SA), systemic acquired resistance (SAR), a kind of broad-spectrum disease resistance that develops throughout the whole plant, is activated [11]. PAMPs may also contribute to SAR initiation in *Arabidopsis* [12].

For diseases caused by necrotrophic pathogens that derive nutrients from dead or dying cells and hemi-biotrophic pathogens that obtain nutrients from both living and dead tissues, R gene-mediated HR is only beneficial for their growth, and therefore alternative defense mechanisms exist. Jarosch et al. [13] reported that barley (*Hordeum vulgare* L.) plants carrying the *mlo* mutant gene that confers a durable resistance against powdery mildew are hypersusceptible to the hemi-biotroph rice blast fungus *Magnaporthe grisea*. A gene conferring resistance to the *Puccinia coronata* biotroph in oat (*Avena sativa*) controls susceptibility to the *Cochliobolus victoriae* necrotroph [14]. It has been speculated that toxins or damage to host cells generated by necrotrophic pathogens may initiate defense responses [15]. A number of studies have demonstrated that jasmonic acid (JA) and ethylene (ET) signaling pathways play important roles in resistance against necrotrophic pathogens and hemi-biotrophic pathogens. Mutation of the JA receptor protein *COI1* gene alters resistance to necrotrophic pathogens such as *Alternaria brassicicola* and *Botrytis cinerea* [16]. Similarly, *Arabidopsis* ET insensitive mutants, such as *etr1* and *ein2-1*, are susceptible to necrotrophic pathogens [17]. The ERF1 and ORA59 transcription factors integrate these two pathways and activate expression of defense-related genes such as *PDF1.2* [18], [19]. In addition to the JA/ET pathways, signaling pathways mediated by heterotrimeric G proteins, such as those involving ABA and DELLA proteins, show positive effects to necrotroph resistance [20]–[22].

The mechanisms underlying resistance to necrotrophic and hemi-biotrophic pathogens are complicated. The SA and JA/ET defense pathways generally interact antagonistically in the resistance response. Many mutants compromised in SA signaling are susceptible to biotrophic pathogens and display an enhanced resistance to some, if not all, necrotrophic pathogens. Mutants compromised in JA/ET signaling usually show the contrary [23]. However, exceptions to these findings have been reported in a number of studies. For example, SA signaling is not required for resistance of *Arabidopsis* to *Verticillium longisporum* [24], but is essential for the resistance to the soil-borne pathogenic *Pythium irregulare* and *Fusarium oxysporum* oomycetes [20], [25]. The latter has a lifestyle and infection strategies similar to *V. longisporum*. Cell death lesions resulting from reaction oxidative species (ROS) are associated with *F. oxysporum* infection in the *Arabidopsis cpr5/hys1* mutant [26], but with growth restriction of *F. oxysporum* f. sp. *Asparagi* in asparagus [27]. *Arabidopsis* resistance to *A. brassicicola* is not affected by disturbance of ET signaling, but requires JA signaling [28]. These results suggest that resistance to non-biotrophic pathogens might be host-pathogen specific.

Most current knowledge regarding plant disease resistance derives from studies of dicotyledonous species (e.g. *Arabidopsis thaliana*), with little knowledge having been derived from monotyledonous plants such as rice, wheat, maize, and sorghum. This discrepancy not only limits our understanding of plant resistance strategies, but also limits our capacity to curb diseases affecting agricultural productivity. Here, we investigated the molecular response of wheat to *F. graminearum* attack, with the aim of identifying key resistance-associated genes and the molecular events governing early resistance reactions.

Like *F. oxysporum*, *F. graminearum* Schwabe [teleomorph *Gibberella zeae* (Schweinitz) Petch] is a ubiquitous filamentous fungus. It is the main causal agent of head blight or ‘scab’ in wheat (*Triticum*), barley (*Hordeum*), rice (*Oryza*), and oats (*Avena*), and *Gibberella* stalk and ear rot disease in maize (*Zea*). Fusarium species also cause root rot, seedling blight, and foot rot [29]. Fusarium head blight (FHB), or ear rot, occurs mainly in areas with high humidity during flowering, and has emerged as the most serious wheat and barley disease in China, North America, and Europe. Wheat FHB epidemics often result in 10–15%, and sometimes even >50%, yield loss [30]. Furthermore, the presence of mycotoxins including nivalenol and deoxynivalenol (DON) in the infected grains are detrimental to both humans and livestock [31]. Nganje et al. [32] estimated that the direct and secondary economic losses due to FHB for all crops in the Northern Great Plains and Central United States amounted to $2.7 billion from 1998 to 2000 alone. Because of the serious threat to food security and safety imposed by scab disease, improving scab resistance of wheat cultivars has become a major focus in wheat genetics and breeding. Although a great deal of effort has been made to genetically improve disease resistance, the progress has been slow and inefficient because of its complex genetic nature.

*F. graminearum* can infect cereal florets through natural openings and by direction penetration of the epidermal cuticle and cell wall. It produces various hydrolyzing enzymes to facilitate penetration [29]. Cell wall reinforcement, accumulation of plant defense compounds, and a higher transcription level of xylanase inhibitors, chitinase, glucanase, and pathogenesis-related (PR) genes have been associated with the attack response [33]–[36]. Some of the early signaling events associated with infection of dicot plants by necrotrophic pathogens, such as oxidative burst and scavenging, and JA/ET dependent defense signaling were also related to the *F. graminearum*-wheat interaction [37]–[39]. However, their exact roles and the coordinated regulations in FHB resistance have yet to be well defined. A few studies have shown that a cautious approach must be taken in extrapolating data from dicot plants to cereals. For example, the *Arabidopsis PR1* gene is only responsive to SA treatment, while its wheat homolog *PR1.1* is only responsive to methyl JA treatment [40]. Although disruption of SA signaling in dicots could enhanced resistance to some necrotrophic pathogens, over-expression of *Arabidopsis NPR1* enhanced FHB resistance in wheat, likely together with faster and stronger SAR activation [41].

Here we focused on the clarification of the early signaling events in wheat responding to *F. graminearum* by employing an integrated proteomics and transcriptomics approach and expression profile investigation of genes involved in these events in FHB-resistant Wangshuibai and its susceptible mutant. We demonstrated that there is a biphasic signaling event that involving Ca^2+^, SA, and JA/ET pathways and their coordinated and ordered activation are critical to the occurrence of the resistance. These findings are helpful to shed light on mechanisms underlying resistance to hemi-biotrophic pathogens in monotyledonous plants, and to provide critical clues to curb crop diseases caused by *Fusarium* species.

## Results

### Contrasting phenotypes in resistance to *F. graminearum* infection and spread within spikes

**Figure 1:**
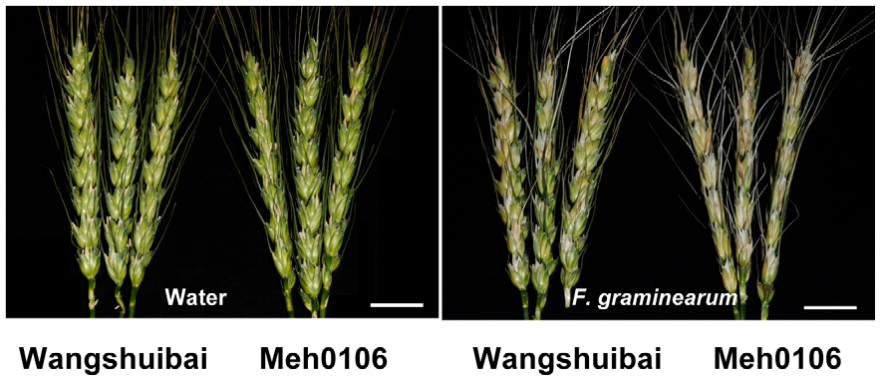
Disease symptoms of Wangshuibai and the susceptible Meh0106 mutant 15 d after *F. graminearum* inoculation, compared with water-mimicked treatment. Scale bar represents 2.5 cm.

In this study, we employed two lines with contrasting response to *F. graminearum* infection. As shown in the phenotyping study, fifteen days after spraying inoculation, only small chlorotic/necrotic lesions confined to the infection points of the spikes were noted in Wangshuibai, a germplasm with a high level of FHB resistance. However, the FHB-susceptible Meh0106 mutant displayed severe disease symptoms in the inoculated spikes, with chlorosis and necrosis reaching almost the entire inoculated spikes (Figure 1). The compromised resistance of Meh0106 to infection and the spread of *F. graminearum* within Meh0106 spikes were validated in repeated trials (Table 1).

**Table 1:** Disease development in Wangshuibai and the susceptible Meh0106 mutant 15 d after inoculation.

Line	NDS		LDR (cm)		PDS (%)		PIS (%)	
	2006	2007	2006	2007	2006	2007	2006	2007
Wangshuibai	1.01***	0.99***	0.09***	0.11***	4.94***	4.31***	17.30*	16.97*
Meh0106	4.89	4.47	2.84	2.34	28.21	25.79	51.83	53.59

***P = 0.001;

**P = 0.01;

*P = 0.05.

### Distinct protein profiles after infection with *F. graminearum*

**Figure 2:**
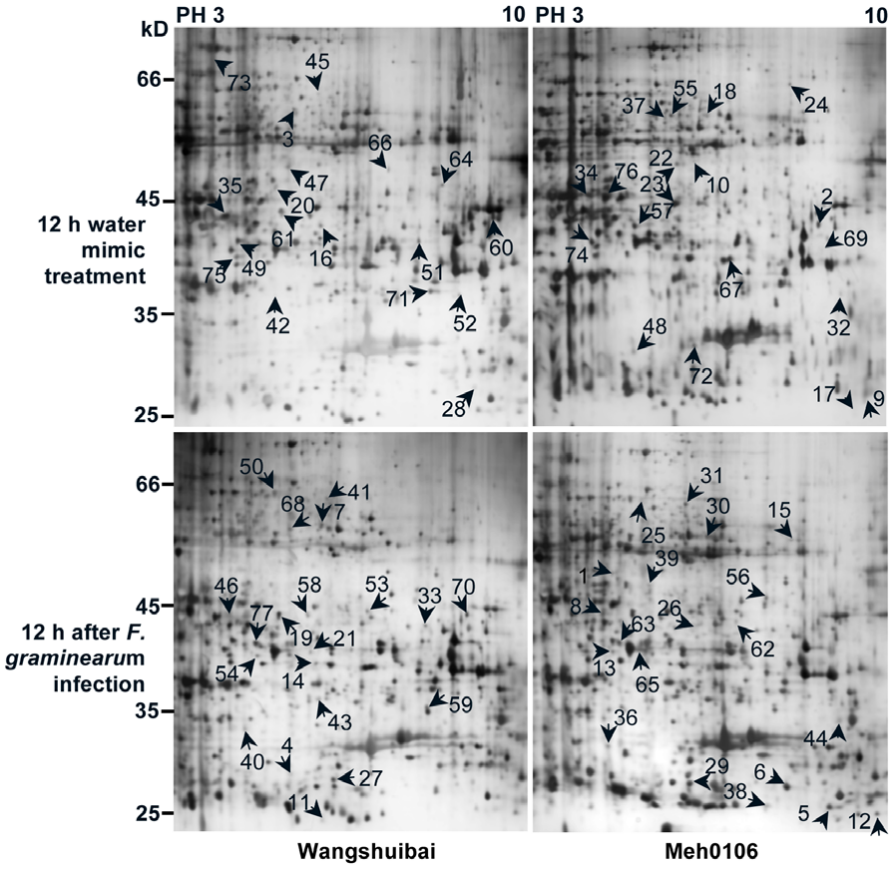
Silver-stained 2-DE of proteins extracted from Wangshuibai and Meh0106 spikes at 12 hai with *F. graminearum* or H_2_O. This is a representative image from three technical and three biological replicates.

**Figure 3:**
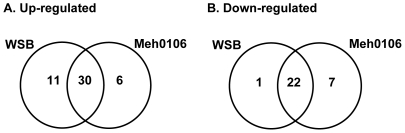
Venn diagram indicating number of proteins differentially expressed in Wangshuibai (WSB) and Meh0106 after *F. graminearum* infection. A. Number of up-regulated proteins; B. Number of down-regulated proteins.

To investigate the proteome-level responses of scab-resistant Wangshuibai and FHB-susceptible Meh0106 plants to initial infection of *F. graminearum*, we compared their protein profiles in spikes 12 h after spraying inoculation (hai). Total proteins were subjected to 2-DE analysis with 17-cm gel strips (Figure 2). In three independent experiments, over 600 protein spots were detected reproducibly after silver staining, mostly distributed from 20 to 100 kDa with pI values in the range of 4 to 9. Compared to the mock treatment, there were 77 protein spots showing at least 1.5-fold volume changes in one or both of the genotypes, more than 70% of which exhibited at least 2-fold changes (Figure S1). Although most spots showed quantitative changes, seven of them showed qualitative changes after infection, of which four were only detected in the infected spikes in both lines (spots 12, 17, 36, 40), one was detected in the infected mutant (spot 38), and two were only detected in spikes without inoculation (spots 47, 73). There were 47 spots up-regulated (Figure 3A) and 30 down-regulated by the infection (Figure 3B), of which a total 52 (67%) showed similar variation patterns between Wangshuibai and the mutant (Figures 3A and 3B). It was noticeable that 25 spots showed up- or down-regulation only in one of the two lines, of which 12 (19%) spots showed line-specific volume changes in Wangshuibai, with one down-regulation spot and 13 (20%) spots showed line-specific volume changes in the mutant. These results implied that substantial cellular events in response to *F. graminearum* infection occur in both Wangshuibai and the mutant.

### Protein identities of spots showing volume changes responding to *F. graminearum* infection

Protein identities for 60 of the 77 spots were determined by querying their MALDI-TOF MS data against the *Triticeae* peptide database (Tables 2 and S1). Five spots (spots 6, 20, 28, 47, and 52) were matched to proteins without any functional information. Nine up-regulated Wangshuibai-specific spots had determined protein identity: four are proteins for the defense signal molecules in ET or JA biosynthesis, four are proteins related to disease defense reactions, and one is related to stress response. Five up-regulated mutant-specific spots had determined protein identity: one is for lysine synthesis that is beneficial for fungal growth, and three are involved in protein degradation. 22 spots showed up-regulation in both Wangshuibai and the mutant, and 18 have been associated with disease resistance or defense (Tables 2 and S1). Among them, spots 12 and 41 are for basal resistance, spots 67 and 71 are related to ET synthesis, spots 1 and 39 are for ROS production, and spots 11, 46, and 48 are for antioxidant defense. These results implied that JA/ET defense signaling and the ROS production and scavenging system are key players in Wangshuibai scab resistance.

**Table 2:** Summary of regulated proteins detected in Wangshuibai (WSB) and the susceptible mutant Meh0106.

Functional category		Upregulated			Down-regulated			Total
		Specific		Common in both lines	Specific		Common in both lines	
		WSB	Meh0106		WSB	Meh0106		
Defense-related	JA/ET biosynthesis	4	/	2	/	/	/	6
	Antimicrobial compound synthesis or detoxification	1	/	3	/	/	/	4
	Antioxidative stress	/	/	5	/	/	5	10
	Cell morphogenesis or cell wall fortification	1	/	1	/	4	/	6
	Other defense related	3	/	7	1	/	/	11
Not associated with defense	Protein degradation	/	3	/	/	/	/	3
	Amino acid synthesis	/	1	/	/	/	/	1
	Other non–defense related	/	/	2	/	2	10	14
	Unknown	/	1	2	/	/	2	5
Total		9	5	22	1	6	17	60

Spot 60 was the only down-regulated Wangshuibai-specific spot and was identified as a 2OG-Fe(II) oxygenase family protein. One member protein of the 2OG-Fe(II) oxygenase family in *Arabidopsis* is required for susceptibility to downy mildew [42]. Of the six down-regulated mutant-specific spots with determined protein identity, four are related to cell morphogenesis or cell wall formation, implying that disturbance of cell wall structure compromised mutant scab resistance. Among the 17 down-regulated spots occurring in both Wangshuibai and the mutant with determined protein identity, five might be related to reduction of oxidative burst. The remaining spots are mostly related to photosynthesis or energy metabolism.

### Comparative transcriptome identification of genes differentially expressed in response to *F. graminearum* infection

To more comprehensively understand the wheat response to *F. graminearum* infection, we identified up-regulated genes by examining changes at the transcriptome level using an *in silico* strategy. Up-regulated genes may include genes expressed only after pathogen attack and those expressed in higher abundance after pathogen attack. To identify genes in the former category, we compared 20,878 ESTs from libraries prepared with young wheat spikes (flowering tissues) inoculated with *F. graminearum* to 1.4 million ESTs from libraries prepared with different tissues of wheat, barley, and other *Triticeae* species not affected by biotic stress using a cutoff of ≥90% homology in at least 100 bp sequence overlap, or ESTs from other cereal plants not affected by biotic stress deposited in the NCBI EST database using a cutoff of ≥80% homology. After removing poor quality sequences and duplicated copies, we obtained 696 ESTs that were only expressed after the pathogen attack. Using 90% homology as the cutoff, these ESTs were assembled into 533 unigenes including 117 contigs and 416 singletons. Functional annotations were obtained for 113 of them through BLASTx similarity searches.

To identify genes in the second category, all wheat ESTs except those in the first category were grouped using a cutoff of <90% homology. From the 85,051 groups generated by this process, 5,015 that had at least two ESTs from the *F. graminearum*-induced libraries (ESTs from SSH libraries prepared with *F. graminearum*-infected tissues were not counted) were subjected to further analysis. In each of these groups, we counted the ESTs from the *F. graminearum*-induced libraries (excluding the SSH ones) and those from libraries prepared with flower tissues not subjected to *Fusarium* inoculation at stages starting from meiosis to 24 hai post-pollination. The latter included a total of 25 libraries and 115,795 ESTs. ESTs bearing the same clone name were counted once. By assuming each group represented one unigene, we used the EST frequency of occurrence in the flower tissue libraries without pathogen attack as the estimate of its normal expression rate. The probability of sampling a certain number of ESTs in a specific group from the *F. graminearum*-induced libraries was then calculated. One hundred and thirty nine unigenes were considered to be up-regulated, since they had twice the respective EST frequency from the induced libraries than the frequency in each of the non-induced libraries and also had <0.001 sampling probability. The homologs of these unigenes in other cereal plants were also expressed less abundantly/frequently in the corresponding non-stressed flowering tissues. Functional annotations were obtained for 106 of them through BLASTx similarity searches.

**Figure 4:**
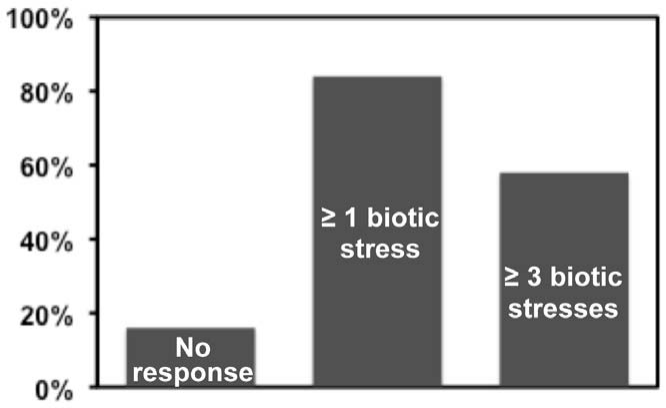
Percentage of the *Arabidopsis* homologs up-regulated by biotic stresses.

Of the 219 up-regulated unigenes with annotation, 163 (74%) were functionally associated with defense against pathogen attack (Tables 3 and S2). We then examined the expression profiles of 55 *Arabidopsis* homologs using the microarray data from treatments of six types of biotic stresses downloaded from Bio-Array Resource (http://www.bar.utoronto.ca/). Most of them, 46/55 (84%), had a higher expression level under at least one kind of biotic stress, and 32/55 (58%) had a higher expression level under at least three kinds of biotic stresses (Figure 4). These results confirmed that the strategy used for identification of genes associated with response to *F. graminearum* infection was effective.

**Table 3:** Summary of genes induced by *F. graminearum* infection as detected by *in silico* Northerns.

Functional category		Number of genes
Defense-related	SA biosynthesis or SA signaling	8
	JA/ET biosynthesis or JA/ET signaling	10
	PA biosynthesis or PA signaling	3
	Cross-talk of signaling pathways	3
	Antimicrobial compound synthesis or detoxification	35
	Antioxidative stress	15
	Resistance gene analogs or kinase proteins	29
	Ca^2+^ signaling	4
	Post-transcriptional regulation	6
	Genes for cell wall fortification	4
	Other defense related genes	46
**Not associated with defense**		56
Total		219

Of the 163 up-regulated defense related unigenes, 117 were classified into ten functional categories based on their putative functions (Tables 3 and S2). Many of the remaining 46 functionally un-classified unigenes are JA/ET-inducible and thus could be placed into some of these categories as more information becomes available.

Eight of the up-regulated unigenes (IGS001–IGS008) are related to SA defense pathways, of which seven code for PR1, PR2, and PR5, the SAR marker genes in dicot plants. IGS002 codes for a R2R3 Myb-like protein, which is associated with HR and programmed cell death (PCD) regulation in response to pathogen attack through induction of SA biosynthesis [43]. Ten unigenes (IGS009–IGS018) were associated with JA/ET biosynthesis or JA/ET signaling: IGS009 codes for JASMONATE INSENSITIVE 1-like protein and IGS016 codes for AtPFT1-like transcription coactivator, both of which are required for JA-dependent defense signaling [44], [45]; IGS017 codes for a ERF1-like protein, where ERF1 is a positive regulator of disease resistance responses in the JA/ET pathways [18]. IGS010 and IGS018, coding for short-chain alcohol dehydrogenase and iron/ascorbate-dependent oxidoreductase, respectively, might be related to JA and ET biosynthesis [20], [46]. It is noteworthy that there were three unigenes (IGS019–IGS021) related to PA signaling and four (IGS104–IGS107) related to Ca^2+^ signaling, suggesting their involvement in the response to *F. graminearum* infection. IGS022 and IGS023 encode BRI1-associated receptor kinase (BAK1)-like proteins that are associated with integration of diverse perception events into downstream PAMP responses leading to systemic immunity [47]. IGS024 encodes a MAP kinase phosphatase (MKP) whose homolog in *Arabidopsis* is a negative regulator of the MAPK cascade, repressing ICS1 mediated SA biosynthesis through calmodulin (CaM) binding [48], implying that they were components for fine-tuning of the SA signaling pathway. Thus, these three unigenes are related to crosstalk of signaling pathways. Transcriptome analysis also revealed fifteen disease resistance gene analogs (RGAs) (IGS075–IGS089) and fourteen protein kinases (IGS090–IGS103). Strikingly, most of these unigenes were expressed only after pathogen attack. They could be important for regulation of crosstalk between signaling pathways mediated by phytohormones in response to pathogen infection [49].

Other up-regulated unigenes include 35 (IGS025–IGS059) that encode proteins involved in antimicrobial compound synthesis or detoxification, 15 (IGS060–IGS074) that encode proteins involved in anti-oxidative stress, 6 (IGS108–IGS113) that encode proteins associated with defense-related post-transcriptional regulation, and 4 (IGS114–IGS117) that encode cell wall modifying enzymes. Interestingly, 16 of the unigenes related to antimicrobial compound synthesis or detoxification, and almost all the unigenes related to defense-related post-transcriptional regulation were found selectively within spikes under attack, suggesting that *Fusarium* infection directly activated defense-related secondary metabolism and a complicated signaling network regulated at multiple levels.

### Defense-related signaling pathways were expressed differently in Wangshuibai and the susceptible mutant

Transcriptomic analysis showed that wheat responded to *F. graminearum* infection by turning on several defense-related signaling pathways, including those mediated by SA, JA, ET, Ca^2+^, and PA. Proteomic analysis also showed that the proteins related to JA/ET signaling were expressed differentially between Wangshuibai and susceptible mutant plants. Here, we examined the time-course expression profiles of key genes involved in these pathways through quantitative real-time reverse transcriptase PCR (qRT-PCR) of samples collected from infected flowering tissues of Wangshuibai and the susceptible Meh0106 mutant to verify these findings and to examine the crosstalk between different pathways.

#### SA signaling pathway

**Figure 5:**
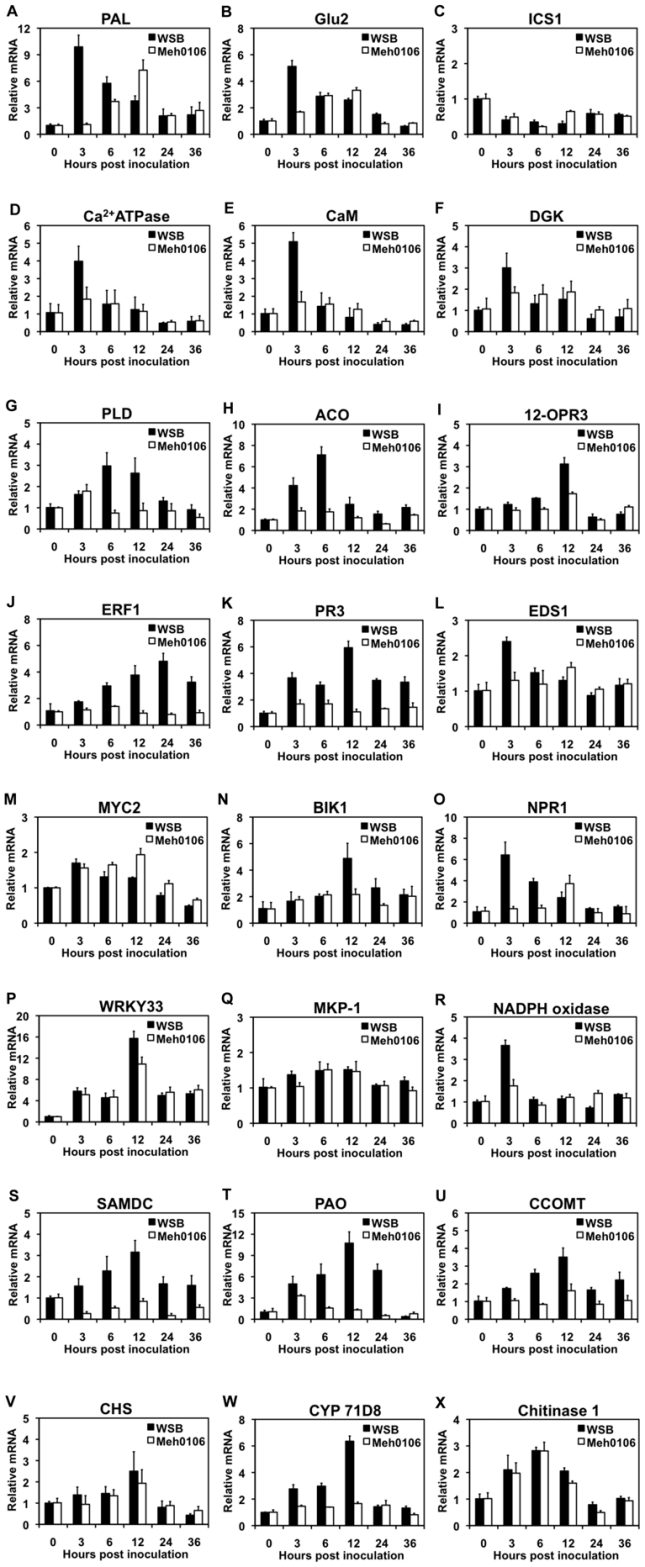
Expression profiles of *PAL* (A), *Glu2* (B), *ICS1* (C), Ca^2+^ *ATPase* (D), *CaM* (E), *DGK* (F), *PLD* (G), *ACO* (H), *12-OPR3* (I), *ERF1* (J), *PR3* (K), *EDS1* (L), *MYC2* (M), *BIK1* (N), *NPR1* (O), *WRKY33* (P), *MKP-1* (Q), *NADPH oxidase* (R), *SAMDC* (S), *PAO* (T), *CCOMT* (U), *CHS* (V), *CYP71D8* (W) and *Chitinase 1* (X) in Wangshuibai (WSB) and Meh0106 spikes after *F. graminearum* infection. The expression levels were relative to no inoculation (0 h) after normalization of the qRT-PCR outputs with the wheat *tubulin* gene output. RT-PCR was performed using gene-specific primers (Table S4). The experiment was repeated three times with similar results. Data were presented as average + S.D with n = 3.

To see how the SA signaling pathway, which governs local resistance and SAR, reacted to *F. graminearum* infection, we investigated the expression of phenylalanine ammonia lyase gene (*PAL*), isochorismate synthase gene (*ICS1*), and β-(1,3; 1,4)-glucanase-2 (*Glu2*) within 36 hai. PAL is the key enzyme for SA biosynthesis via the phenylpropanoid pathway [50]. Wildermuth et al. [51] reported that, in *Arabidopsis*, most SA is synthesized via the isochrorismate pathway, employing ICS1 as the key enzyme. *ICS1* homologs have since been identified in a number of plants including wheat. In the qRT-PCR analysis, we noted that *PAL* transcription was induced rapidly in Wangshuibai, peaking within 3 hai, while *PAL* induction in the susceptible mutant was markedly slower and less efficient (Figure 5A). The expression profiles for *Glu2*, which responds exclusively to SA signaling [40], were the same as those for *PAL* (Figure 5B). Meanwhile, *ICS1* was down-regulated after *F. graminearum* infection and did not differ considerably between Wangshuibai and the mutant (Figure 5C). Thus, the *F. graminearum* infection caused increased PAL, but not ICS1, expression, which could result in SA accumulation via the phenylpropanoid pathway. These results suggest that early activation of SA signaling could be a critical factor in scab resistance and that a delay in activation of this pathway may be associated with susceptibility.

#### Ca^2+^ signaling pathway

Calcium-transporting ATPase is a major regulator of intracellular Ca^2+^ concentrations in support of proper cell signaling. We investigated the expression profiles of the differentially expressed IGS104 that encodes a calcium-transporting ATPase-like protein and the wheat homolog of the rice calcium-transporting ATPase 1. Though the RT-PCR of IGS104 was not successful, the calcium-transporting ATPase 1 homolog showed significant transient increases at 3 hai in both resistant and non-resistant plants (Figure 5D), even though to a much greater degree in the resistant plants. The wheat homolog of maize calmodulin1, a key regulatory protein in Ca^2+^ signal transduction, exhibited the same expression pattern as the calcium-transporting ATPase 1 homolog (Figure 5E). These results imply that the Ca^2+^ signaling pathway participated in the early reaction of wheat to *F. graminearum* infection and is related to basal resistance. Hence, the mutant plant' basal defense may be compromised.

#### PA signaling pathway

IGS019 encoding diacylglycerol kinase (DGK) and IGS020 encoding phospholipase D (PLD) were only identified in the *F. graminearum*-infected flowering tissues. DGK and PLD are key enzymes in different phosphatidic acid (PA) synthesis pathways. PA is a lipid second messenger in plants whose involvement in biotic and abiotic stress responses is gaining attention. *DGK* was rapidly activated after infection in Wangshuibai and that *PLD* was activated about 3 hai later and a little more efficiently (Figures 5F and 5G). Their expression patterns were consistent with pathogen-induced biphasic PA accumulation, first via the PLC-DGK pathway then via the PLD pathway [52]. In the mutant, *DGK* and *PLD* induction were not efficient (Figures 5F and 5G) and the slight change in their expression levels might suggest a lack of PA accumulation. The DGK expression profile was similar to that of PAL (Figures 5A and 5F), which is not surprising since DGK is inducible by the SA analog benzothiadiazole (BTH) [53]. By SA addition to *Arabidopsis* cell suspensions, Krinke et al. [54] demonstrated that PLD activation is also an early component of the SA signaling pathway. Thus, the association of the PA signaling pathway with scab resistance may have resulted from SA signaling, and be involved in PLC/DGK-mediated signaling in oxidative burst and hypersensitive cell death [53].

#### JA/ET signaling pathway

Both transcriptomic and proteomic analyses showed that the JA/ET pathways were actively involved in the response to *F. graminearum* infection. We found that the gene for 1-aminocyclopropane-1-carboxylic acid oxidase (ACO) (spot 33) enzyme catalyzing the oxidation of ACC to ethylene, and the gene for jasmonate biosynthesis isoenzyme 12-oxophytodienoate reductase 3 (12-OPR3) (spot 58) were both only elevated in Wangshuibai (Table S1). Similarly, the induction was only noted in this resistance material in the time course studies of their expression (Figures 5H and 5I). Maximum *12-OPR3* expression occurred around 12 hai. Compared with *12-OPR3, ACO* was activated more rapidly, with the transcripts peaking around 6 hai.

To ascertain the roles of JA and ET pathways in scab resistance, the *PR3* marker gene for JA/ET-mediated defense response and the wheat ERF1 homolog were examined. ERF1 is a key element in the integration of JA and ET signaling [18]. The transcript level of the ERF1 homolog showed a gradual increase after infection in Wangshuibai that became significant at 3 hai (Figure 5J). *PR3* was induced slightly in Wangshuibai after *F. graminearum* infection before 6 hai, and then peaked at 12 hai (Figure 5K). The expression levels of both genes remained unchanged in the mutant within 36 hai (Figures 5J and 5K). These results clearly supported the view that there is an association between the JA/ET pathways and scab resistance.

#### Interactions between SA and JA/ET pathways

Since SA defense pathways are generally known to be antagonistic to the JA/ET pathways, to investigate their coordination in responding to *F. graminearum* infection we checked the temporal transcript changes of a few SA regulatory genes including *EDS1*, *MYC2*, *BIK1*, *NPR1*, *MPK4*, and *WRKY33*. *EDS1* is an essential component of R gene mediated disease resistance in *Arabidopsis* and is required for SA-regulated basal resistance by modulating SA accumulation [55]. *MYC2* is a pathogen-inducible gene and negatively regulates the expression of JA/ET pathway-associated defense genes in *Arabidopsis* by acting upstream of ERF1 [21]. *BIK1* negatively regulates SA accumulation and has a positive effect on the expression of the JA-responsive *PDF1.2* gene [56]. *NPR1* positively regulates SA signaling and induces PR gene expression by nuclear localization after pathogen attack [57]. Cytosolic *NPR1* has a negative effect on JA signaling [58]. *MPK4* and *WRKY33* are components of the MAPK cascade. The former negatively regulates the SA pathway and positively regulates the JA/ET pathway through *EDS1/PAD4* [59]. The latter is an *in vitro* substrate of *MPK4* and has a similar regulatory role as *MPK4* on the SA and JA/ET defense pathways in response to hemi-biotrophic and necrotrophic pathogens [60].

As shown in Figure 5L, *EDS1* was weakly induced at 3 hai in Wangshuibai. The transcript then declined by 6 hai and reached a non-infection level by 12 hai. The *MYC2* homolog had a similar expression pattern (Figure 5M). Interestingly, their declines were coincident with a gradual increase in *BIK1* transcript levels that peaked at 12 hai (Figure 5N). The *NPR1* expression profile was similar to that of *EDS1*, except that it had a much more efficient induction at 3 hai (Figure 5O). In the mutant, *EDS1, MYC2*, and *NPR1* had similar expression profiles. They showed slight induction at 3 hai, but the induction continued and peaked at 12 hai. In contrast, although *BIK1* had the transcription efficiency similar to that of *EDS1* at 3 hai in the mutant, it did not show significant induction at 12 hai relative to 6 hai. These results suggest that appropriate defense reactions to *F. graminearum* attack at different time frames depend on proper activation of particular pathways. The mutant's susceptibility was caused, at least in part, by failed timely positive regulation of the JA/ET pathway due to reduced function of *BIK1* and inappropriate high levels of *NPR1* and *MYC2*. Higher *EDS1* and *NPR1* activity at 12 hai in the mutant may indicate a higher level of SA signaling that is unfavorable to the needed JA signaling.

In contrast to *BIK1*, *WRKY33* had maximum abundance at 12 hai in both Wangshuibai and the mutant (Figure 5P), although the mutant expression level was lower. Similar expression patterns were also observed for IGS024, which encode MKP1-like protein (Figure 5Q), and *MPK4* (data not shown). This finding suggests that though the MAPK cascade may be involved in responses to *F. graminearum* infection, but is not the factor responsible for the differential resistant responses between Wangshuibai and the mutant.

### ROS production and the subsequent anti-oxidative activity differed between Wangshuibai and the susceptible mutant

Transcriptomic and proteomic analyses revealed that genes and proteins involved in ROS production and relieving oxidative stress were induced after infection. It is well known that ROS, including the superoxide radical anion (O_2_^−^), hydroperoxyl radical (HO_2_•), hydroxyl radical (HO•), and hydrogen peroxide (H_2_O_2_) accumulate during the earliest events in many plants under pathogen attack. However, the role of ROS is far clearer against biotrophic pathogens than against necrotrophic and hemi-necrotrophic pathogens. Since H_2_O_2_ is relatively stable among ROS, we examined the association with *F. graminearum* infection of the polyamine oxidase (PAO) pathway and the NADPH oxidase pathway that play critical roles in H_2_O_2_ production in plants [61], [62]. In the PAO pathway, S-adenosylmethionine decarboxylase (SAMDC) catalyzes polyamine biosynthesis, which is then converted to H_2_O_2_ by PAO. In the NADPH oxidase pathway, NADPH oxidase generates superoxide. Superoxide dismutase (SOD) then dismutates superoxide into oxygen and H_2_O_2_. A transient increase in NADPH oxidase transcripts, which peaked at 3 hai, was observed in Wangshuibai (Figure 5R), while transcripts of SAMDC and PAO showed a gradual increase in Wangshuibai before 24 hai (Figures 5S and 5T). We noted negligible change in the expression levels of these genes in the susceptible mutant, suggesting that ROS production is related to resistance to *F. graminearum* infection.

**Figure 6:**
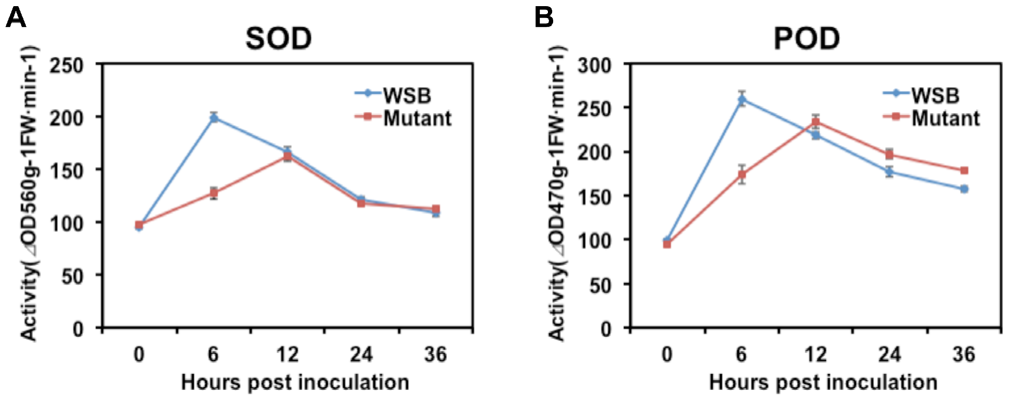
Activities of SOD (A) and POD (B) after *F. graminearum* infection in Wangshuibai and Meh0106 spikes. Each data point consisted of three replicates. Error bars indicate SD. WSB: Wangshuibai; Mutant: Meh0106.

Consistent with the induced expression of NADPH oxidase gene, in Wangshuibai, the activities of SOD and peroxidase (POD), which break down H_2_O_2_, more than doubled in the first 6 hai (Figures 6A and 6B). Relative to those in Wangshuibai, the activity changes in the mutant were slower and less prominent.

### Genes for cell wall modification and biosynthesis of antimicrobial compounds were differentially expressed in Wangshuibai and the susceptible mutant

In addition to the defense signaling pathways, components in defense-related metabolic pathways were also identified at the proteome and/or transcriptome level, such as cell wall fortification, antimicrobial compound biosynthesis, and PR gene activation. Expression of caffeoyl-CoA 3-O-methyltransferase (CCOMT) (spot 36), a critical in lignin synthesis and functions in structural barrier defense [6], was almost unchanged in the mutant, but was induced as early as 3 hai in Wangshuibai, peaking 12 hai. Moreover, CCOMT showed a relatively higher level in Wangshuibai than in the susceptible mutant throughout the infection response process (Figure 5U).

With respect to antimicrobial compound biosynthesis, we investigated the expression profiles of IGS057 encoding chalcone synthase (CHS) and IGS031 encoding cytochrome P450 71D8 (CYP71D8), which catalyze biosynthesis of flavonoid type phytoalexins and camalexin type phytoalexins, respectively [63], [64]. Induction of IGS057 was similar in Wangshuibai and the mutant (Figure 5V). However, IGS031 was only significantly induced in Wangshuibai, peaking at 12 hai (Figure 5W), indicating that synthesis of camalexin type phytoalexins could be important for FHB resistance. Chitinase 1, associated with the protein represented by spot 09, degrades fungal cell walls [65]. The transcription of *Chitinase 1* was up-regulated after *F. graminearum* infection and no significant difference in expression was observed between Wangshuibai and the susceptible mutant (Figure 5X). Like IGS057, Chitinase 1 may participate in the defense response to FHB, without contributing to resistance.

### SA and JA contents changed differentially in Wangshuibai and the susceptible mutant after the infection

**Figure 7:**
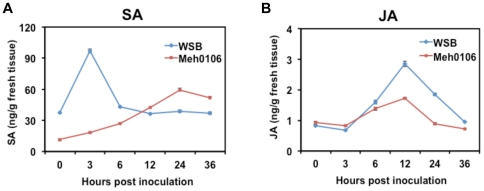
Endogenous contents of SA (A) and JA (B) in Wangshuibai and Meh0106 spikes at different time points after *F. graminearum* infection. Each data point consisted of three replicates. The experiments were repeated twice with similar results. Error bars indicate SD.

Since SA and JA signaling are all involved in reactions to *F. graminearum* infection, the endogenous contents of SA and JA in tissues used in the expression analysis were investigated. The variation patterns of their concentrations across the time points were remarkably similar to the expression profiles of the investigated genes corresponding to the respective pathways (Figure 7). The SA concentration in Wangshuibai increased 3-fold at 3 hai, but declined rapidly at 6 hai; its rise in the susceptible mutant occurred slower and reached the highest point at 24 hai (Figure 7A). In both Wangshuibai and the mutant, the induced JA accumulation started at 3 hai and reached the peak levels at 12 hai (Figure 7B). Interestingly, the SA content was in a much lower basal level in the mutant than in Wangshuibai, implying that the mutant have SA synthesis deficiency.

## Discussion

In the mutant library created with the FHB-resistant Wangshuibai, we identified the Meh0106 mutant that has completely lost FHB resistance. Substantial alternation by mutation of phenotypes governed by QTLs has been reported in other plants as well. For example, the rice *moc1* mutant has only the main culm with no tillers [66]; *moc1* is a recessive mutation of MOC that encodes a putative GRAS family nuclear protein regulating the control of tillering. Given the disruption of multiple defense pathways in Meh0106, it is also likely that the loss of resistance is due to mutation of a regulator critical to FHB resistance in Wangshuibai. Measurement of the SA and JA contents in Wangshuibai and the mutant revealed that the latter has much less basal SA. SA is one of most important regulating molecules participating in disease resistance in plants.

In recent years, a number of genes or proteins have been identified through analyzing the expressed products of genes after *F. graminearum* infection [37]–[39], [67]–[71]. Their functional annotations suggested that multiple events occurred in the defense response. Nevertheless, little is known about the molecular events contributing to the contrasting performances of resistant and susceptible genotypes to *F. graminearum* infection because of different genotypes used, limited resolution of the technologies, small size of the investigated cDNA and protein samples, and particularly the lack of genetically different materials only in the resistance-related genes. The availability of Meh0106 made it feasible to explore the molecular mechanisms governing FHB resistance.

### Proteome and transcriptome profiling indicated that multiple defense-related signaling and cellular events contribute to FHB resistance

By comparing the spike proteomes of Wangshuibai and its FHB-susceptible mutant without *F. graminearum* infection with those at 12 hai of *F. graminearum*, we identified 77 protein spots showing at least 1.5-fold volume change from over 600 protein spots. Although the number might not be extraordinary, our annotation results are quite informative. Most of the up-regulated proteins, either from the resistant Wangshuibai or from the susceptible mutant or from both, are involved in basal resistance or defense (Tables 2 and S1). These results were not different from other similar studies [38], [69]. The present study is distinguished from others by the identification of proteins involved in JA and ET synthesis and of proteins related to the phenylpropanoid pathway. The lack of up-regulated PR proteins identified in this study can most likely be attributed to the fact that we used a 12-h time interval between inoculation and sampling, while others used at least a 24-h time interval [38], [69]. Hence, activation of the JA/ET pathway may be a relatively early defense event and the massive production of PR proteins a relatively late defense event. It is noteworthy that four of the nine up-regulated spots found only in Wangshuibai were proteins associated with either ET or JA biosynthesis and one was associated with defense-related lignification. Meanwhile, the six down-regulated spots found only in the mutant were predominantly proteins related to cell morphogenesis or cell wall formation, implying that JA/ET defense signaling and cell wall modification are important resistance reactions in FHB resistance.

Transcriptome analyses of resistant and susceptible genotypes after *F. graminearum* infection using suppression subtractive hybridization, microarray, and cDNA-RFLP revealed that SA, JA/ET signaling, antioxidative reactions, and defense-related secondary metabolism were associated with defense reactions [37], [70], [71]. However, the identified gene numbers and profiles differ substantially between studies due to differences in the genotypes and technologies used, and thus do not constitute a comprehensive description. Moreover, in Li and Yen [37], more than half of the 608 genes identified as associated with the defense response could not be functionally annotated. In the present study, we did *in silico* transcriptome comparison of the *F. graminearum* infected tissues with the corresponding healthy tissues, regardless of the genotypes of the compared ESTs. This resulted in findings that were consistent with our proteome analysis that enabled identification of extra signaling and cellular events contributing to defense reactions. Although only 219 of the 639 identified pathogen responsive genes could be annotated, three quarters of those identifications were functionally defense-related. Notably, besides the findings in the proteome analysis, multiple components of the SA, PA, and Ca^2+^ signaling pathways, as well as the MAPK cascade, were up-regulated by pathogen infection. Moreover, a number of genes for RGAs and kinase proteins, post-transcriptional regulation, and secondary metabolism such as antimicrobial compound synthesis and detoxification were only found in spikes under pathogen attack, implying their unique roles in defense against *F. graminearum*. Through expression profiling, we confirmed the involvement of the identified individual pathways and cellular events in scab resistance (Figure 5). A few other studies have also reported that some genes for cytochrome P450 proteins, UDP-glucosyltransferase, PR proteins, JA/ET biosynthesis, and RGAs are differentially expressed in resistant genotypes [37], [68], [71]. RGAs and kinase proteins have been related to crosstalk regulation between signaling pathways mediated by plant hormones in response to pathogen infection. For example, R protein Mi-1 mediated aphid resistance in tomato involves both SA and JA signaling [49]. Differentially expressed NB-ARC domain containing RGAs were also expressed differentially in comparisons between the transcriptomes of a pair of wheat near-isogenic lines carrying either the resistant or susceptible allele at the FHB-resistant QTL *Fhb1* after infection [39].

Some of the identified genes did not display differential expression patterns between resistant and susceptible genotypes, such as *CHS* (IGS057) and *chitinase 1* (spot 09) (Figures 5V and 5X), suggesting that they are probably involved in general defense reactions. This phenomenon seems to be common among biotic stress-related genes [33], [36]. Moreover, even though *WRKY33, MPK4*, and the *MKP1*-like gene (IGS024) of the MAPK cascade were induced by *F. graminearum* infection, they were not associated with the mutant's susceptibility. Overexpressing these genes might still be useful for enhancing resistance [72].

### The early defense reactions to *F. graminearum* infection had a biphasic strategy

For resistance to biotrophic pathogens, SA is the central player in HR and SAR, while Ca^2+^, nitric oxide, and PA are also associated with early signaling events [9], [11], [73], [74]. They work together to trigger oxidative burst and activate a specific set of PR genes. For resistance to *F. graminearum*, an arguably hemi-biotrophic pathogen [67], [75], the expression profiles of SAR associated markers, such as *PR1*, *PR2*, and *PR5*, after the pathogen infection indicate that SAR is part of the early defense reaction [33], [36], [72]. In wheat, faster activation of the defense response and significant enhancement of FHB resistance occurred when expressing *AtNPR1*, the key positive regulator of *Arabidopsis* SAR [41]. Genes involved in the SA pathway, including *PAL*, *EDS1*, *NPR1*, and *Glu2*, were rapidly induced in Wangshuibai (Figures 5A, 5B, 5L and 5O). Their maximum expression occurred at 3 hai and declined thereafter. The induction in the mutant was much slower and less substantial. The change patterns of SA content after the infection in both lines were consistent with the expression profiles of these genes. Moreover, the mutant has less basal SA. These results suggested the importance of SA signaling pathway in scab resistance and the SA synthesis deficiency in the mutant might be the causal factor of its susceptibility. Recently, Makandar et al. [75] and Cuzick et al. [76] reported that SA signaling through *NPR1* is important for limiting disease severity caused by *F. graminearum* and *F. culmorum* in *Arabidopsis*. These results all support the role of SA in FHB resistance. However, Pritsch et al. [36] and Li and Yen [37] reported conflicting results. They noted that the SAR marker genes were induced by pathogen infection, but showed no difference in expression between the resistant and susceptible genotypes. The requirement for SA signaling is similar to the reaction of *Arabidopsis* to infection of *F. oxysporum* [25], which is consistent with the initial biotrophic process of both pathogens. It is likely that SA signaling is an early general defense response or part of the innate immune reaction to *F. graminearum* infection regardless of genotype, but is also an important prerequisite for later resistance development. Nevertheless, it should be noted that SA signaling is not required for resistance to all hemi-biotrophic pathogens [24]. Plants can synthesize SA via PAL or ICS1 [50], [51]. However, only PAL was responsive to *F. graminearum* infection. This observation is similar to that in *Arabidopsis* plants, which synthesize SA via the PAL pathway in response to the *B. cinerea* necrotrophic fungi [77].

Ca^2+^ signaling is critical for transcriptional reprogramming in plant innate immunity [9]. The variation in cytosolic Ca^2+^ concentration upon pathogen infection mediates the signaling process. A few studies have demonstrated that a transient change in Ca^2+^ permeability of the plasma membrane is a common early event key to plant defense signaling [78]. In the response to *F. graminearum* infection, the examined calcium-transporting ATPase 1 gene and CaM gene were rapidly but transiently induced, similar to the SA signaling-related genes (Figures 5D and 5E). The transcripts also diminished rapidly after 3 hai, which is consistent with the observation that there is rapid but transient Ca^2+^ accumulation in the cytoplasm during the initial plant-pathogen interaction stage [9]. The differences between the Wangshuibai and mutant expression profiles are striking. Therefore, we concluded that both SA and Ca^2+^ signaling are primary concurrent signaling events important for the occurrence of resistance.

It is widely accepted that the JA signaling pathway mediates resistance to necrotrophic and hemi-biotrophic pathogens. The findings of the current study and of Li and Yen [37] support this notion. The JA content increased at 3 hai and in a much faster rate in the FHB-resistant Wangshuibai than in the mutant (Figure 7B). In accordance with this, all the tested JA signaling-related genes, including *12-OPR3* (spot 58), *ERF1*, and *PR3*, were induced strongly in Wangshuibai, but induced only weakly in the mutant (Figures 5I, 5J and 5K), indicating that the JA pathway was suppressed to some extent in the susceptible mutant. This interpretation is consistent with the expression profile of *BIK1*, which acts as a negative regulator of SA accumulation and a positive regulator of the JA/ET response [56], and was not turned on in the mutant at 12 hai as in Wangshuibai. The significantly higher level of *MYC2* expression in the mutant at 12 hai might also not favor timely activation of the JA defense pathway, as *MYC2* plays a negative regulatory role in the JA-mediated defense response against *F. oxysporum* in *Arabidopsis* [21]. Unlike the Ca^2+^ and SA signaling-related genes, maximum induction in Wangshuibai occurred at 12 hai, suggesting that activation of the JA signaling pathway followed activation of the Ca^2+^ and SA defense pathways.

Interestingly, the expression of *DGK* (IGS019) and *PLD* (IGS020) also had a biphasic expression pattern (Figures 5F and 5G), with *DGK* being induced concurrently with the SA and Ca^2+^ pathway genes, and *PLD* induced almost concurrently with the JA pathway genes. Both genes contribute to PA synthesis and are SA-inducible [53], [54]. *PLD* is also required for wound-induced JA biosynthesis [79]. These expression profiles suggest that *DGK* expression may be part of the SA signaling pathway while *PLD* expression may be associated with pathogen-induced JA biosynthesis, although no evidence is available to support this assertion. There is a biphasic ROS burst in response to avirulent pathogen infection [3] and PA can promote elicitor-induced biphasic ROS burst in rice suspension cells [52]. Concurrent with the above-mentioned biphasic patterns, the expression of NADPH oxidase gene was rapidly induced, peaking at 3 hai, while the expression of *SAMDC* and *PAO* was more slowly induced, peaking at 12 hai (Figures 5R, 5S and 5T). All three genes encode key enzymes for H_2_O_2_ production. Lherminier et al. [80] showed that the first wave of H_2_O_2_ production is mediated by NADPH oxidase after challenge of tobacco cells with fungal elicitor cryptogein. The differential gene expressions between Wangshuibai and the mutant suggest that they are all part of an integrated resistance system.

In Wangshuibai, the induced expression of NADPH oxidase was accompanied by rapid elevation of the activities of the enzymes POD and SOD (Figures 6A and 6B), both of which are ROS scavengers. There are also active antioxidative activities after infection, as shown in the omics analyses. We speculate that ROS generation is precisely controlled, which benefits FHB resistance since there is only a transient biotrophic stage in *F. graminearum* infection [81] and the subsequent PCD caused by ROS could be favorable for *F. graminearum* growth. After *F. graminearum* infection, the plants might use multiple strategies to control PCD. Early induction of calcium-transporting ATPase 1 could help control PCD [82]. NADPH oxidase could also participate in cell death suppression at sites surrounding its activation, thus restricting the spread of PCD [83]. Their low level expression in the mutant could result in uncontrolled PCD, contributing to the susceptibility. Moreover, Kemmerling et al. [84] reported that *BAK1*, which is induced after the infection, has a role in controlling infection-induced PCD. The second phase accumulation of ROS generation enzymes concurred with the expression of JA signaling genes. There is evidence that ROS may be required for activating and establishing JA/ET signaling [85]. ROS also takes part in lignin polymerization, which is a resistance-related cell wall modification event when attacked by *F. graminearum* [86].

In summary, the sequential induction of endogenous SA and JA accumulations and the altered expression peak time of signaling pathway genes points to adoption of a biphasic strategy in the early wheat defense reactions to *F. graminearum* infection, with the initiation of Ca^2+^ and SA signaling preceding JA signaling. This strategy may be a feature of plant resistance to some hemi-biotrophic pathogens. PA and ROS accumulation accompanied these two phases, but probably through different synthetic pathways. We noted significant positive correlations between the expression profiles of *PAL*, *CaM*, *NADPH oxidase*, and *DGK* in Wangshuibai (Table S3), which imply a close association between the initial events. This relationship was not observed in the mutant. Ca^2+^ can positively activate PA production, which in turn is implicated in increasing NADPH oxidase activity and ROS production [87]. Thus, Ca^2+^ signaling could play a positive regulatory role in SA signaling and the initial ROS burst in the first phase of FHB resistance reaction. The ROS burst could result from SA signaling or provide positive feedback to the SA defense pathway [88], [89]. It is likely that, in the susceptible mutant, mutation of a factor having a similar role as Ca^2+^ signaling or being part of Ca^2+^ signaling *per se* disrupted the initial phase of defense signaling events, resulting in failed activation of ET signaling and the subsequent activation of JA signaling.

### The two-phase resistance reactions are well coordinated to ensure the successful occurrence of resistance

We have shown that both SA and JA signaling are associated with FHB resistance. However, these two defense-signaling pathways are known to have an antagonistic relationship. Thus, it would be interesting to examine how they are coordinated in the resistance-related early molecular events. We noted that all expression profiles of the examined genes that positively regulate SA signaling were induced regardless of genotype. However in Wangshuibai, the induction peak was at 3 hai, while in the mutant it was at 12 hai, at the same time as maximum expression of JA signaling-related genes. In addition, the maximum transcript levels were significantly higher in Wangshuibai than in the mutant. Similarly, there was also a common pattern for the expression profiles of the examined genes that positively regulate JA signaling. That is, all were induced in Wangshuibai with their peaks at 12 hai and their induction was attenuated in the mutant. The sequential induction of SA and JA content increase would be an effective coordinative mechanism of the two antagonistic pathways, and the delayed induction of SA accumulation in the mutant disrupts this coordination, which results in susceptibility. Indeed, induction of SA and JA signaling concurrently promoted disease severity caused by *F. graminearum* in *Arabidopsis* [75]. We postulated that timely and orderly activation of the SA and JA defense pathways is critical in order for them to coordinately confer resistance. Consistent with this notion, exposure of *Arabidopsis* to MeJA at the beginning of *F. graminearum* infection enhances disease severity, while exposure to MeJA at 12 and 24 hai increases resistance [75]. The expression patterns of all crosstalk genes, except those linked to MAPK cascade, and of SA and JA signaling genes provide further evidence supporting this hypothesis. Their maximum expressions occurred at the same time as the expression of genes positively regulated by them, and were almost at background (without infection) levels at the same time as the maximum expression of genes negatively regulated by them (Figures 5L–5Q).

The association of ET synthesis with failed mutant resistance is intriguing. ET signaling has been positively related to FHB resistance in the FHB-resistant Sumai 3 cultivar [37]. We found that the ET biosynthesis gene *ACO* (spot 33) had maximum expression at the time point between the maximum expression of SA and JA signaling related genes. This intermediate presence could be an important regulatory step in the transition from SA mediated defense to JA mediated defense, since ET signaling can render JA response insensitive to SA antagonism in *Arabidopsis* [90]. Moreover, the activation of the SA signaling pathway may be a prerequisite for optimal induction of ET synthesis since ozone-induced ET accumulation is compromised in NahG and *npr1* plants [91]. *ERF1/ORA59* could play a regulatory role in the transition process. The gradual increase in *ERF1* expression level in Wangshuibai after infection corresponds to its function (Figure 5J). In susceptible germplasm, this could be a different scenario, since ET signaling could be exploited by *F. graminearum* and thus increase susceptibility [92]. Different branches of ET signaling might be involved in the resistance/susceptibility reactions, since even though the key ET biosynthesis *ACO* gene and the downstream ET signaling *ERF1* gene showed differential expression between Wangshuibai and the mutant after infection, the expression of *EIN2*, a key component of the ET pathway, was not altered with infection (data not shown). However, caution should be taken since changes in the ET level could influence EIN2 protein stability in the absence of any effect on *EIN2* mRNA levels [93].

### FHB resistance is associated with cell wall reinforcement, antimicrobial compound synthesis, and detoxification

Appropriate activation of early defense signaling events leads to disease resistance, which is implemented by cellular activities such as synthesis of phytoalexins, detoxification enzymes, and cell wall modifications. In the omics analyses, a number of genes related to these activities were induced after *F. graminearum* infection. Even though the Meh0106 mutant phenotype likely resulted from the mutation of a regulatory gene, it does not present with differential expression of all resistance implementation genes. For example, the expression of *CHS* (IGS057) (Figure 5V) and *CYP99A1* (IGS030) (data not shown) that are associated phytoalexin biosynthesis in plants, and of *chitinase 1* (spot 09) (Figure 5X) with the function of degrading fungus cell wall, was induced in both Wangshuibai and the mutant after infection, suggesting that they are involved in basal defense but not resistance. On the other hand, significant induction of *CYP71D8* (IGS031) and *CCOMT* (spot 36) was observed in Wangshuibai but not in the mutant (Figures 5W and 5U), indicating their association with resistance. *CYP71D8* contributes to production of the phytoalexin camalexin, which is involved in resistance to necrotrophic pathogens in *Arabidopsis* [64]. *CCOMT* contributes to lignin biosynthesis, a phenolic cell wall polymer associated with plant defense against biotic and abiotic stresses and indispensable for plant structure and defense [6]. In an FHB-resistant cultivar, lignin content in the cell wall increased at a higher rate than in susceptible cultivars [94]. UDP-glycosyltransferase that is inducible by *F. graminearum* infection detoxifies the DON toxin produced by the fungus and may be involved in the *Fhb1*-associated FHB resistance [95], [96].

### A putative regulatory network for FHB resistance

**Figure 8:**
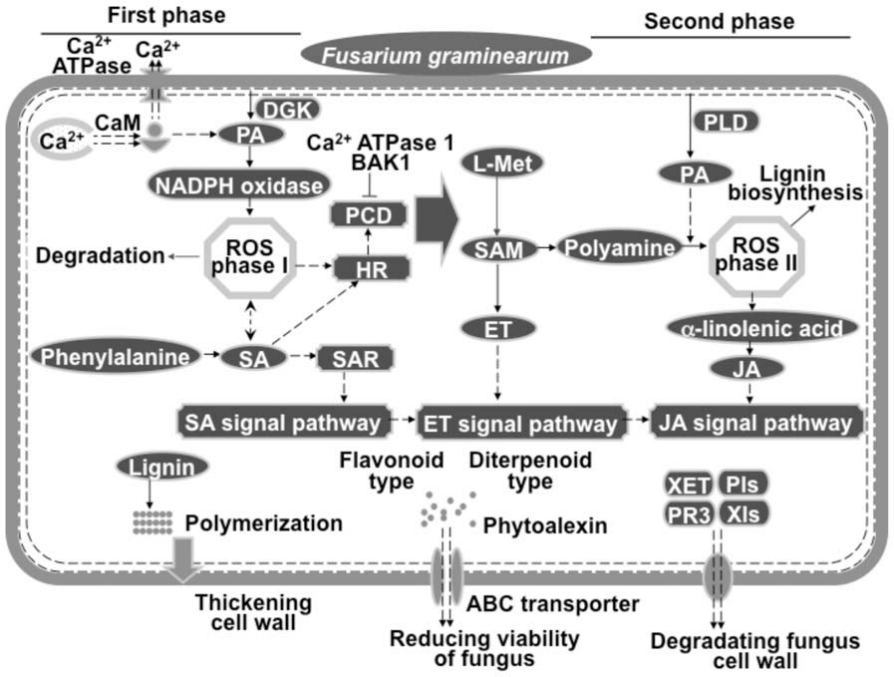
A model illustrating pathways leading to FHB resistance. PIs, protease inhibitors; XET, xyloglucan endotransglycosylase; Xls, xylanase inhibitors. See the text for other abbreviations. The peripheral solid lines indicate cell wall, the peripheral dotted lines indicate plasma membrane; the solid arrows represent direct interactions, and the dotted arrows represent indirect interactions. Perpendicular line on arrows indicates negative relationship.

We have demonstrated that resistance to hemi-biotrophic *F. graminearum* infection is associated with coordinated and ordered expression of diverse defense signaling pathways and altered secondary metabolism. Based on the content change patterns of defense signaling molecules as well as the sequential events and differential expression profiles of the involved genes in Wangshuibai and the susceptible mutant after *F. graminearum* infection, we propose a model to illustrate the early cellular events leading to FHB resistance (Figure 8). There were two major phases of resistance reactions during the first 24 hai. The first phase occurred within 6 hai, probably corresponding to a transient biotrophic stage. In this stage, changes or activation of Ca^2+^ fluxes, Ca^2+^ signaling, SA signaling, PA signaling, and ROS production and scavenging were the major cellular activities. Since they are all related to HR and PCD and in order to constrain cell death that favors *F. graminearum* growth, multiple mechanisms controlling PCD were also activated during this stage. The second phase occurred after 6 hai and before 24 hai, probably corresponding to the start of the necrotrophic stage. In this stage, the JA/ET signaling pathway and ROS production via PAO were activated, which lead to a series of resistance reactions. ET signaling activation occurred between the two major phases, facilitating the transition from SA to JA defense signaling, since they are usually antagonistic. The activating order of these signaling events could be critical in forming resistance. Crosstalk genes, such as *EDS1*, *NPR1*, *BIK1*, and *ERF1*, were actively involved in the regulation processes. The resistance related activities such as PR protein production, cell wall enforcement, and antimicrobial compound synthesis and detoxification could commence soon after infection and peak 6 to 12 hai. Both SA and JA/ET defense signaling pathways can activate these related genes [63], [96]. We have identified a few genes for resistance-related cell modification and phytoalexin production that could be exploited as a worthy strategy for improving resistance against *F. graminearum* infection.

## Materials and Methods

### Plant materials and spike inoculation

Wangshuibai is a common indigenous wheat germplasm of Jiangsu, China. ‘Meh0106’ is a homozygous FHB-susceptible mutant obtained from M_2_ progenies of Wangshuibai with dry seeds treated with 0.35% (w/v) EMS. They are similar in phenotype except for their resistance level to *F. graminearum*.

To evaluate resistance to pathogen penetration, spikes at anthesis were inoculated by spraying, and then water was sprayed to maintain moisture. Percentage of diseased spikelets (PDS) and percentage of infected spikes (PIS) were investigated 15 d after the inoculation [97]. To evaluate resistance to pathogen spreading, spikes at anthesis were inoculated through point inoculation, and the number of diseased spikes (NDS) and the length of diseased rachides (LDR) were investigated 15 d post-inoculation in 2006 and 2007 [98]. The inocula were composed of four local virulent strains of *F. graminearum* (F4, F15, F17, and F34) at a concentration of about 1000 conidiospores per 25 µl. Inoculated spike tissues were harvested at 3, 6, 12, 24, and 36 hai. The control was prepared by mimicking the inoculation with pure water. Tissues for proteomic analysis were prepared in a similar way.

### Protein extraction

Proteins were extracted from young spikes of Wangshuibai and Meh0106 according to Damerval et al. [99]. Concentration was determined using Bradford's method with bovine serum albumin (BSA) as the standard [100].

### 2-D electrophoresis and gel staining

One-hundred-and-twenty-microgram aliquots of protein samples were included in 350 µL rehydration buffer (7 M urea, 2 M thiourea, 32.5 mM CHAPS, 0.5% (v/v) carrier ampholytes pH 3 to 10, 65 mM dithiothreitol and 0.002% (w/v) bromophenol blue). IPG strips (17 cm, pH 3 to 10, nonlinear; Bio-Rad, Hercules, California, USA) were passively rehydrated with rehydration buffer containing the protein sample for 13 h in the Protean system (Bio-Rad). IEF was performed at 250 V for 1 h, 500 V for 1 h, 10 000 V for 2 h, 4000 V for 2 h, all in a linear gradient, and then maintained at 8000 V until at least a total of 56 000 Vh was reached. After IEF, the strips were equilibrated in equilibration buffer I (6 M urea, 69 mM SDS, 0.05 M Tris–HCl, pH 8.8, 20% (v/v) glycerol, 65 mM dithiothreitol) at ambient temperature for 15 min, and then in equilibration buffer II (6 M urea, 69 mM SDS, 0.05 M Tris–HCl, pH 8.8, 20% (v/v) glycerol, 135 mM iodoacetamide) for another 15 min. After the equilibration, the strips were positioned on top of the 2nd-dimension gel and sealed with 1% (w/v) agarose for SDS-PAGE. The 10% SDS-polyacrylamide gels were run in the Ettan DALTtwelve vertical electrophoresis system (Amersham Biosciences, Freiburg, Germany) at 40 mA for 30 min followed by 60 mA for 4.5 h. Silver staining was carried out according to Shevchenko et al. [101]. This experiment was performed with three biological replicates each with three technical triplicates.

### Image analysis

The stained gels were scanned with Gel Doc 3000 (Bio-Rad) and the images were analyzed using PDQuest software (Bio-Rad). After alignment, automatic spot matching was performed. The matched spots were then examined manually and falsely matched or unmatched spots were corrected. The quantity values of the spots were exported after detection, standardization, and background elimination. Spots with a quality value over 50 and showing 1.5-fold change in abundance between treatments were selected for further analysis.

### In-gel digestion

De-staining, reduction and iodoacetamide treatments, and trypsin (Promega, Madison, WI, USA) digestion of the selected spots were carried out as described in Shevchenko et al. [101]. Peptides were then extracted from gel pieces once by 5% (w/v) TFA and twice by 2.5% (w/v) TFA in 50% (v/v) acetone (each for 1 h) at 37°C. The resulting peptides were lyophilized completely in a vacuum centrifuge and dissolved in 2 µL 0.5% (w/v) TFA.

### MS analysis and protein identification

The peptide solution was mixed with the supernatant of 60% ACN (Fisher Scientific, Springfield, NJ, USA) saturated with a-cyano-4-hydroxycinnamic acid (Sigma, St. Louis, MO, USA), and then air dried on the flat surface of a sample plate. MS analysis was carried out with the MALDI-TOF mass spectrometer Reflex III (Bruker-Daltonics, Billerica, MA) in the positive ion reflector mode and analyzed by the peaklist-generating software FlexControl™ 2.2. Calibration was carried out using autolytic tryptic peptides. The mass spectra data were collected from mono isotopic peaks falling in the m/z range of 750 to 4000 Da with S/N ratio over 10. Outputs resulting from autolysis of trypsin and from commonly occurring keratin contamination were excluded from the subsequent data query.

Using a customized searching tool based on eMowse from EMBOSS (http://emboss.sourceforge.net/), peptide mass fingerprint (PMF) data were matched to wheat polypeptide database constructed from 1,046,811 wheat EST sequences using *cTrans* [102], using the setting of monoisotopic molecular weight, missed cleavage = 1, mass tolerance = 50 ppm, fixed modification = carbamidomethyl (Cys) and variable modification = oxidation (Met). ESTs corresponding to the peptide fragments were used to search ESTs with at least 40 base overlap and 94% sequence homology in *Triticeae* EST database to construct cDNA contigs. Final sequence coverage was calculated by comparing the m/z values obtained by MALDI-TOF MS with the peptide fingerprints obtained by *in silico* trypsin digestion of the conceptual proteins translated from the contigs using Masspeptide program (http://www.expasy.org). The criteria for a successful identity determination were: at least 3 matched peptides and 20% sequence coverage; isoelectric point differing by at most 2; and molecular weight differing by at most 20% compared with the experimental estimate.

### *In silico* transcriptomic analysis

Extraction of EST sequences from the GenBank database (ftp://ftp.ncbi.nih.gov/genbank/) and vector or adaptor contamination removal were carried out with software *cTrans*. Fungal DNA contamination was removed by querying the DNA databases of fungi *Fusarium graminearum*, *Fusarium oxysporum*, *Botrytis cinerea*, *Magnaporthe grisea*, *Sclerotinia sclerotiorum*, *Stagonospora nodorum*, *Ustilago maydis*, *Puccinia graminis*, and *Pyrenophora tritici repentis* (http://www.broad.mit.edu/annotation/fgi/). All sequence comparisons were carried out with the BLAST tools provided by National Center for Biotechnology Information (NCBI) [103]. CAP3 was used for sequence assembly [104]. The probability to obtain a certain number of ESTs for a gene under the induced condition in the given library size was estimated using the equation 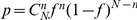, where N = total number of ESTs, n = number of ESTs corresponding to the target gene, f = expression frequency of the target gene under the normal condition. The identified genes were annotated based on Gene Ontology (GO) and information identified in literatures, and classified according to their biological functions.

### Microarray data analysis

Homologs of the wheat genes in *Arabidopsis* were identified through BLASTxing against the *Arabidopsis* protein database (http://www.arabidopsis.org/) without low complexity filtering, using the threshold of E<1E-4 and >70% similarity. Microarray data produced in six kinds of biotic stresses (http://www.bar.utoronto.ca/) including infections of *Botrytis cinerea*, *Pseudomonas syringae*, *Phytophtora infestans*, and *Erysiphe orontii*, and treatments of bacterial- and oomycete-derived elicitors, were employed to explore their association with biotic stresses. A homolog was considered to be up-regulated if its peak expression level under a specific biotic stress was at least 1.5-fold higher than the control. Here the expression level was an average of repeated microarray experiments at a specific time point.

### qRT-PCR analysis

RNA was extracted using the Trizol reagent (Invitrogen, USA) following the manufacturer's protocol and quantified with a spectrometer (Ultrospec2100 pro, Amersham Pharmacia, England). First-strand cDNA was synthesized with 3.0 µg total RNA in a 20 µl reaction volume using M-MLV reverse transcriptase (Promega, USA) according to manufacturer's instruction. Two µl of 10× diluted cDNA were used as a template for qRT-PCR analysis. PCR reaction was performed in a total volume of 20 µl, containing 2.0 µl cDNA, 250 M of each primer, and 10 µl of 2 x iQ SYBR Green Supermix (Bio-Rad) on an iCycler iQ fluorescence real time PCR (Bio-Rad). The Q-PCR setting was 1 min at 95°C, followed by 40 cycles of 94°C×15 s, 58°C×25 s and 72°C×30 s. Primers (Table S4) were designed according to the wheat cDNA contigs coding for the corresponding proteins. The relative expression level was normalized with the expression data of wheat *tubulin* gene and estimated using the 2^−ΔΔCT^ method of Livak and Schmittgen [105]. The PCR was repeated thrice. There were two biological replicates for PCRs of *NPR1*, *EDS1*, *BIK1*, *PR3*, and *MYC2*.

### SOD and POD assay

Total SOD activity, expressed in units per mg of protein, was assayed according to the method of Beyer and Fridovich [106]. One unit of SOD activity was defined as the sample volume causing 50% inhibition of the absorbance increase, measured at the 560 nm wavelength. POD activity was determined using the guaiacol oxidation method of Chance and Maehly [107]. A unit of peroxidase activity was defined as nmol H_2_O_2_ decomposed per minute per mg of protein.

### Measurement of SA and JA contents

The tissues used in the measurements were the same as those in expression analysis. Extraction and quantification of SA and JA was performed according to Li et al. [108]. Briefly, 200 mg of tissues was extracted with 0.5 mL of 1-propanol/H_2_O/ concentrated HCl (2∶1∶0.002, v/v/v). After centrifugation, the supernatants were loaded on C18 solid-phase extraction cartridges (CNWBOND HC-C18, 500 mg, 3 mL). The elutes were used for HPLC-ESI-MS/MS separation in a HPLC (Agilent 1200, Agilent Technologies, CA) and then quantitation in a hybrid triple quadrupole/linear ion trap mass spectrometer (ABI 4000 Q-Trap, Applied Biosystems, CA) using the multiple reaction monitoring (MRM) and information dependent acquisition (IDA) mode. The standard curves for SA and JA quantification were generated using a series of SA and JA (Sigma) dilutions. These experiments were all performed with two biological replicates and each sample was measured three times.

## Supporting Information

Figure S1 **Histograms showing the volume changes of 77 differentially displayed 2-DE spots.** Data show a representative experiment from three independent experiments with similar results and three replicates each. The error bars indicate SD of three replicates. Y-axis: relative abundance of the protein; X-axis: spot number. a: Wangshuibai 12 hai with H_2_O; b: Wangshuibai 12 hai with *F. graminearum*; c: Meh0106 12 hai with H_2_O; d: Meh0106 12 hai with *F. graminearum*.
(TIF)

Table S1 Protein identities and their functional annotations for the differentially expressed protein spots.
(DOC)

Table S2 Differentially expressed genes after *F. graminearum* infection identified by *in silico* Northerns and their functional annotations.
(DOC)

Table S3 Expression correlations of the genes examined in Wangshuibai and its susceptible mutant Meh0106 involved in the early signaling events after *F. graminearum* infection.
(DOC)

Table S4 Primer sequences used in qRT-PCR.
(DOC)
