# Supplementary figures and images for: Resistance to Hemi-Biotrophic F. graminearum Infection Is Associated with Coordinated and Ordered Expression of Diverse Defense Signaling Pathways
Source: PLoS One. 2011 Apr 20;6(4):e19008. doi: 10.1371/journal.pone.0019008 (PMC3080397; doi:10.1371/journal.pone.0019008)

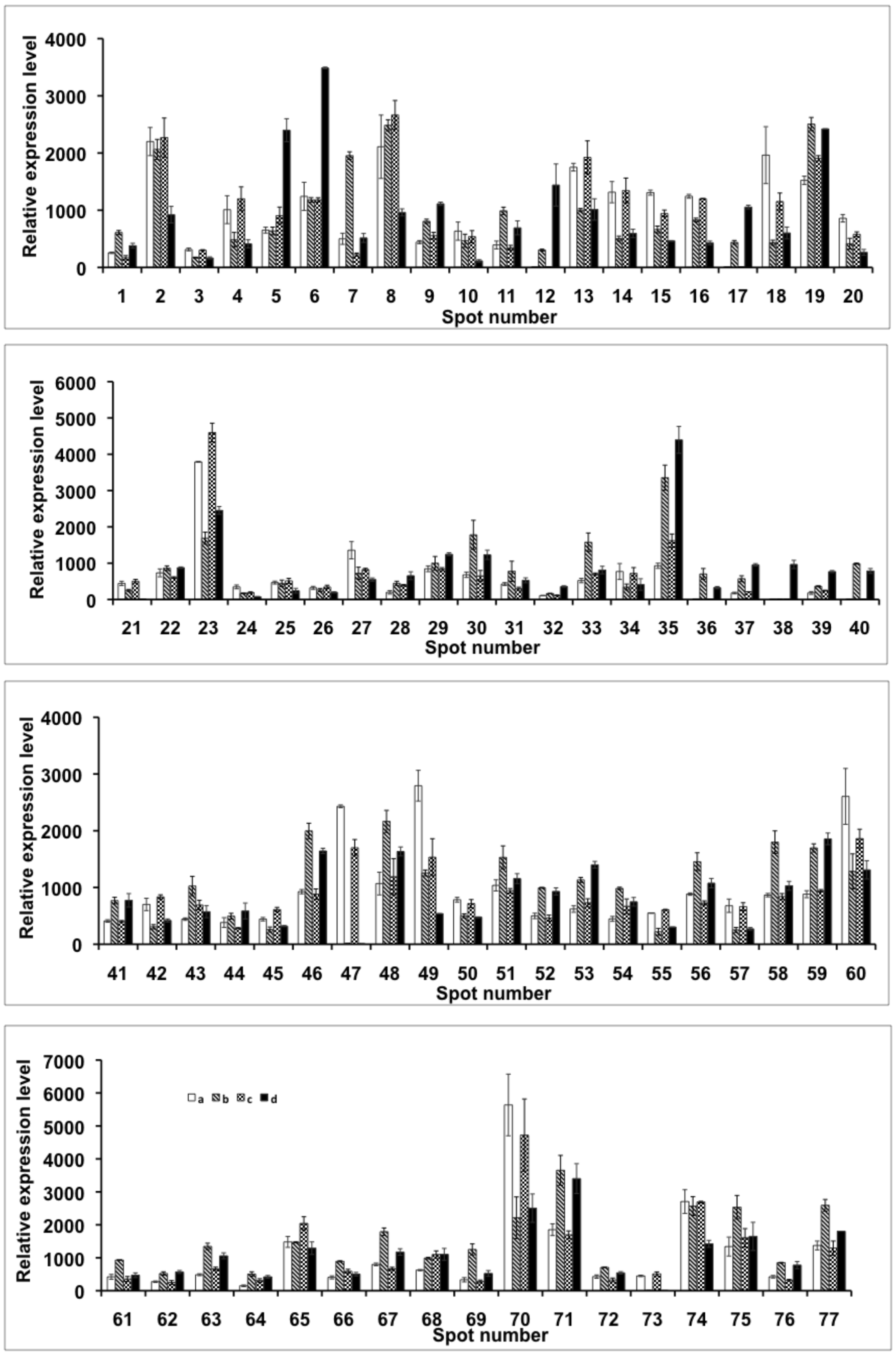

Supplement: Figure S1 — Histograms showing the volume changes of 77 differentially displayed 2-DE spots. Data show a representative experiment from three independent experiments with similar results and three replicates each. The error bars indicate SD of three replicates. Y-axis: relative abundance of the protein; X-axis: spot number. a: Wangshuibai 12 hai with H2O; b: Wangshuibai 12 hai with F. graminearum; c: Meh0106 12 hai with H2O; d: Meh0106 12 hai with F. graminearum. (TIF) [file pone.0019008.s001.tif]
