# Supplementary material for: Resistance to Hemi-Biotrophic F. graminearum Infection Is Associated with Coordinated and Ordered Expression of Diverse Defense Signaling Pathways
Source: PLoS One. 2011 Apr 20;6(4):e19008. doi: 10.1371/journal.pone.0019008 (PMC3080397; doi:10.1371/journal.pone.0019008)
Supplement: Table S1 — Protein identities and their functional annotations for the differentially expressed protein spots. (DOC) [file pone.0019008.s002.doc]

**Table S1. Protein identities and their functional annotations for the differentially expressed protein spots.**

|  | | | | | | | | |
| --- | --- | --- | --- | --- | --- | --- | --- | --- |
| **Spot No.** | **Exp. MW/pI^a^** | **The. MW/pI^b^** | **Coverage (PM)^c^** | **Accession no.** | **Protein name** | **Possible function** | **Fold change^d^**  ***p*-value^e^** | |
| **Up-regulated proteins** | | | | | | | **WSB** | **Meh0106** |
| No.33 | 43.0/6.70 | 35.4/4.98 | 44.97%(9) | BQ295155 | 1-aminocyclopropane-1-carboxylic acid oxidase | Ethylene biosynthesis | 3.0  0.02 | _ |
| No.43 | 36.2/6.00 | 39.3/6.14 | 22.19%(5) | CK164849 | Ethylene-forming enzyme | Ethylene biosynthesis | 2.3  0.03 | _ |
| No.58 | 44.9/5.93 | 42.7/7.48 | 22.31%(5) | CJ628459 | 12-oxophytodienoate reductase 3 | JA biosynthesis | 2.1  0.01 | _ |
| No.61 | 46.4/5.80 | 40.2/5.32 | 33.47%(7) | BQ483977 | 12-oxo-phytodienoic acid reductase 1 | JA biosynthesis | 2.2  0.00 | _ |
| No.54 | 40.4/5.55 | 46.8/5.54 | 24.47%(8) | CK161432 | DnaJ-C chaperone | Hsp40 family protein, defense-related (Byth et al. 2001). | 2.2  0.00 | _ |
| No.64 | 49.0/7.01 | 49.4/7.48 | 40.00%(4) | CJ703979 | GATA transcription factor | Regulating innate immunity in animals (Shapira et al. 2006). | 3.4  0.01 | _ |
| No.51 | 41.9/6.83 | 37.8/5.72 | 24.50%(6) | DR739674 | Aldo–keto reductases | Member of the NAD(P)H oxidoreductases, detoxification (Simpson et al. 2009). | 1.5  0.05 | _ |
| No.66 | 51.0/6.56 | 37.2/4.83 | 45.35%(10) | CJ622728 | Adenosine kinase 2 | Defense-related liginification (Pereira et al. 2007). | 2.2  0.00 | _ |
| No.75 | 40.5/5.38 | 60.6/5.77 | 22.32%(12) | DR739388 | Pyrophosphate-dependent phosphofructo kinase | Restoring cell homostasis following stresses (Danson et al. 2000). | 1.9  0.04 | _ |
| No.29 | 27.7/6.10 | 29.3/5.15 | 29.89%(6) | CA500411 | Diaminopimelate epimerase | Lysine synthesis, while lysine is necessary for hypha growth. | _ | 1.5  0.00 |
| No.05 | 26.5/7.90 | 27.0/7.36 | 31.69%(8) | CJ665292 | Beta 7 subunit of 20S proteasome | Ubiquitin-independent degradation | _ | 2.7  0.00 |
| No.38 | 26.1/6.70 | 29.2/6.19 | 27.65%(4) | DR738777 | 26S proteasome regulatory particle non-ATPase subunit12 | Ubiquitin-independent degradation | / |   0.00 |
| No.44 | 33.7/8.16 | 24.3/6.37 | 57.47%(11) | CJ778656 | Proteasome subunit beta type 1 | Ubiquitin-independent degradation | _ | 2.1  0.05 |
| No.06 | 27.7/6.90 | 26.3/8.23 | 25.82%(5) | CD922255 | Unknown | Unknown | _ | 3.0  0.00 |
| No.09 | 26.6/8.80 | 27.1/9.33 | 46.09%(7) | DN829392 | Chitinase 1 | Degrading fungus cell wall (Abeles et al. 1971). | 1.8  0.00 | 2.0  0.00 |
| No.36 | 31.2/5.50 | 32.1/5.53 | 20.96%(4) | CA629804 | Caffeoyl CoA O-methyltransferase | Lignin biosynthesis, defense response related (Raes et al. 2003). |   0.00 |   0.00 |
| No.63 | 41.3/5.58 | 33.1/5.16 | 35.48%(8) | DR739286 | Isoflavone reductase homolog | Isoflavonoid phytoalexin biosynthesis. | 2.8  0.00 | 1.6  0.02 |
| No.41 | 64.9/6.13 | 55.6/7.19 | 28.26%(3) | CD883027 | Cytochrome P450, similar to CaCYP1 | Basal pathogen defense required. | 1.9  0.00 | 1.9  0.04 |

**Table S1. (continued).**

|  | | | | | | | | |
| --- | --- | --- | --- | --- | --- | --- | --- | --- |
| **Spot No.** | **Exp. MW/pI^a^** | **The. MW/pI^b^** | **Coverage (PM)^c^** | **Accession no.** | **Protein name** | **Possible function** | **Fold change^d^**  ***p*-value^e^** | |
| **Up-regulated proteins** | | | | | | | **WSB** | **Meh0106** |
| No.07 | 58.4/5.90 | 44.9/4.79 | 28.54%(8) | CJ572019 | DNA-damage inducible protein | Disease inducible (Zhou et al. 2006). | 3.9  0.00 | 2.3  0.02 |
| No.35 | 44.5/5.30 | 41.9/5.91 | 24.87%(5) | CJ583034 | O-methyltransferase ZRP4-like | Methyl transfer from S-adenosyl-L-methionine, MeJA induced (Salzman et al. 2005). | 3.6  0.00 | 2.7  0.00 |
| No.32 | 36.1/8.00 | 33.0/9.23 | 34.55%(8) | DR741800 | NAC domain-containing protein, similar to OsNAC6 | Transcriptional regulation of plant development, induced by abiotic signals and JA (Ohnishi et al. 2005). | 1.5  0.00 | 3.3  0.00 |
| No.12 | 25.7/8.90 | 29.0/8.07 | 23.53%(4) | BJ291158 | Polyphosphoinositide binding protein Ssh2p | Lipid transfer regulation, up regulated by flg22 (Chen et al. 2009). |   0.00 |   0.00 |
| No.59 | 35.0/6.88 | 35.6/4.27 | 20.30%(5) | CV768818 | TGB12K interacting protein-3 like | Putative glucanase, disease defense related. | 1.9  0.00 | 2.0  0.00 |
| No.46 | 43.6/5.38 | 35.2/5.63 | 22.05%(5) | CD868700 | Peroxidase | Defense response | 2.1  0.00 | 1.9  0.00 |
| No.48 | 30.9/5.64 | 27.5/5.91 | 46.80%(9) | DR739268 | Ascorbate Peroxidase | Defense response | 2.0  0.02 | 1.5  0.25 |
| No.11 | 25.8/6.00 | 23.4/5.75 | 57.28%(8) | CK217215 | Glutathione transferase F5 | Antioxidant defense enzyme, defense required. | 2.5  0.00 | 2.0  0.04 |
| No.62 | 44.3/6.54 | 41.0/5.61 | 36.15%(9) | CD453630 | Alcohol dehydrogenase III | NADH-dependent reduction of S-nitroglutathione, regulating plant disease resistance (Feechan et al. 2005). | 1.9  0.01 | 2.3  0.01 |
| No.39 | 49.5/5.81 | 35.5/5.69 | 46.25%(15) | CK166703 | Malate dehydrogenase | Producing NADH, early defense related. | 2.0  0.01 | 3.1  0.00 |
| No.01 | 50.0/5.60 | 52.6/5.49 | 29.44%(15) | DR739474 | NADPH producing 6-phosphogluconate dehydrogenase | ROS production in defense reaction (Debnam et al. 2004). | 2.3  0.00 | 2.0  0.02 |
| No.67 | 39.6/6.67 | 42.8/5.42 | 36.55%(9) | CJ786901 | S-adenosylmethionine synthetase 1 | Ethylene biosynthesis | 2.2  0.00 | 1.8  0.00 |
| No.71 | 37.1/6.92 | 42.8/5.42 | 45.43%(12) | CJ786901 | S-adenosylmethionine synthetase 1 | Ethylene biosynthesis | 2.0  0.02 | 2.0  0.02 |
| No.76 | 45.4/5.49 | 48.0/5.35 | 31.84%(12) | [CJ594815](http://genomics.njau.edu.cn/resources/apps/blast/get_seq/query.cgi?cmd=Retrieve&db=Nucleotide&list_uids=93257385&dopt=GenBank) | ENO2_MAIZE Enolase 2 | Production ATP, response to many stresses. | 2.0  0.00 | 2.4  0.01 |
| No.17 | 25.1/8.40 | 24.4/8.26 | 29.30%(7) | BQ245649 | Zinc-binding protein | Transcriptional repression (Nagano et al. 2001). |   0.00 |   0.00 |
| No.30 | 58.2/6.30 | 50.4/5.83 | 25.00%(7) | CJ706262 | Translational elongation factor Tu | Translation | 2.6  0.04 | 1.9  0.04 |
| No.52 | 36.7/7.12 | 24.0/6.18 | 28.70%(5) | CJ653730 | Ras-related protein Rab11C | Unknown | 2.0  0.00 | 2.0  0.00 |
| No.28 | 28.3/7.80 | 25.2/8.88 | 25.23%(5) | CO347917 | Ras_like_GTPase | Unknown | 2.2  0.03 | 1.6  0.04 |

**Table S1. (continued).**

|  | | | | | | | | |
| --- | --- | --- | --- | --- | --- | --- | --- | --- |
| **Spot No.** | **Exp. MW/pI^a^** | **The. MW/pI^b^** | **Coverage (PM)^c^** | **Accession no.** | **Protein name** | **Possible function** | **Fold change^d^**  ***p*-value^e^** | |
| **Down-regulated proteins** | | | | | | | **WSB** | **Meh0106** |
| No.60 | 44.8/8.20 | 44.9/6.95 | 23.33%(5) | CK161252 | Oxidoreductase, 2OG-Fe(II) oxygenase family | A member gene of this family in Arabidopsis negatively affects plant defense (van Damme et al. 2008). | -2.0  0.04 | _ |
| No.08 | 42.8/5.50 | 41.5/5.55 | 35.60%(8) | CK210566 | Reversibly glycosylated polypeptide | Cell wall polysaccharides biosynthesis, defense related (Zavaliev et al. 2010). | _ | -2.8  0.02 |
| No.10 | 52.9/6.20 | 53.1/9.09 | 22.03%(7) | BF202632 | Glycosyl transferase family 8 protein | Cell wall glucuronoxylan biosynthesis. | _ | -4.7  0.02 |
| No.65 | 41.6/5.79 | 35.7/5.87 | 23.75%(7) | BQ608734 | Beta 1,3-glycosyltransferase-like protein II | Cell wall polysaccharides biosynthesis. | _ | -1.6  0.05 |
| No.26 | 42.2/6.20 | 35.5/4.88 | 28.57%(8) | CK207916 | Fructokinase-2 | Starch synthesis | _ | -1.8  0.04 |
| No.74 | 41.0/5.35 | 35.5/5.94 | 21.88%(5) | CD862061 | Peroxisomal targeting signal type 2 receptor | PTS2-directed peroxisomal import ([Woodward and](http://www.ncbi.nlm.nih.gov/pubmed?term="Woodward AW"%5BAuthor%5D&itool=EntrezSystem2.PEntrez.Pubmed.Pubmed_ResultsPanel.Pubmed_RVAbstract) Bartel, 2005). | _ | -1.9  0.00 |
| No.25 | 66.2/5.70 | 66.9/5.96 | 28.45%(12) | CN013133 | Las1-like family protein | Cell growth and morphogenesis ([Doseff and](http://www.ncbi.nlm.nih.gov/pubmed?term="Doseff AI"%5BAuthor%5D&itool=EntrezSystem2.PEntrez.Pubmed.Pubmed_ResultsPanel.Pubmed_RVAbstract) [Arndt, 2005)](http://www.ncbi.nlm.nih.gov/pubmed?term="Arndt KT"%5BAuthor%5D&itool=EntrezSystem2.PEntrez.Pubmed.Pubmed_ResultsPanel.Pubmed_RVAbstract) . | _ | -2.1  0.05 |
| No.15 | 58.3/7.00 | 56.8/6.55 | 32.11%(17) | CJ667791 | CATA1_WHEAT Catalase-1 | Control the equilibrium of antioxidant system. | -2.1  0.00 | -2.2  0.00 |
| No.27 | 28.3/6.10 | 34.3/6.93 | 28.75%(7) | CJ610733 | L-ascorbate peroxidase 6 | Control the equilibrium of antioxidant system. | -1.9  0.05 | -1.5  0.01 |
| No.23 | 45.2/6.00 | 45.9/5.95 | 38.39%(17) | CJ568654 | NADP-specific isocitrate dehydrogenase | ROS production in defense reaction. | -2.2  0.00 | -1.9  0.00 |
| No.21 | 40.1/6.00 | 38.6/6.00 | 37.76%(8) | CK207889 | NADP-dependent oxidoreductase P1 | Plant antioxidant defense, NAD(P)/NAD(P)H homeostasis. | -1.8  0.04 | -43.5  0.00 |
| No.73 | 77.7/5.20 | 80.2/6.39 | 30.15%(17) | CK207938 | NADH-ubiquinone oxidoreductase | Inhibition by both BTH and SA, with superoxide production ([van der Merwe and](http://www.ncbi.nlm.nih.gov/pubmed?term="van der Merwe JA"%5BAuthor%5D&itool=EntrezSystem2.PEntrez.Pubmed.Pubmed_ResultsPanel.Pubmed_RVAbstract) [Dubery](http://www.ncbi.nlm.nih.gov/pubmed?term="Dubery IA"%5BAuthor%5D&itool=EntrezSystem2.PEntrez.Pubmed.Pubmed_ResultsPanel.Pubmed_RVAbstract), 2005. | -  0.00 | -  0.00 |
| No.04 | 27.9/5.80 | 28.6/5.88 | 33.33%(5) | CJ614628 | Ubiquinone oxidoreductase subunit 1 | Electron transfer in mitochondrial respiratory chain. | -2.1  0.05 | -2.9  0.02 |
| No.42 | 36.4/5.67 | 34.4/5.59 | 28.96%(6) | DR740475 | Oxygen-evolving enhancer protein 1 | Photosystem II | -2.3  0.03 | -2.0  0.00 |
| No.57 | 43.4/5.71 | 34.4/5.59 | 33.54%(7) | DR740475 | Oxygen-evolving enhancer protein 1 | Photosystem II | -2.7  0.03 | -2.4  0.01 |
| No.14 | 38.8/6.00 | 36.5/6.77 | 35.91%(11) | CJ577968 | Glyceraldehyde-3-phosphate dehydrogenase | Energy production | -2.6  0.01 | -2.3  0.03 |
| No.24 | 70.2/7.00 | 61.6/5.86 | 24.42%(10) | BJ283147 | Xylulose kinase | Pentose phosphate pathway | -2.0  0.03 | -2.5  0.01 |

**Table S1. (continued).**

|  | | | | | | | | |
| --- | --- | --- | --- | --- | --- | --- | --- | --- |
| **Spot No.** | **Exp. MW/pI^a^** | **The. MW/pI^b^** | **Coverage (PM)^c^** | **Accession no.** | **Protein name** | **Possible function** | **Fold change^d^**  ***p*-value^e^** | |
| **Down-regulated proteins** | | | | | | | **WSB** | **Meh0106** |
| No.16 | 43.2/6.00 | 47.4/6.75 | 33.33%(15) | BE403165 | Aspartate aminotransferase | Nitrogen and carbon metabolism | -1.5  0.00 | -2.8  0.00 |
| No.18 | 60.7/6.30 | 52.7/6.78 | 35.26%(13) | CJ699960 | Dihydrolipoamide dehydrogenase precursor | Photorespiratory | -4.5  0.04 | -1.9  0.04 |
| No.50 | 67.7/5.51 | 52.7/6.78 | 29.88%(8) | CJ699960 | Dihydrolipoamide dehydrogenase precursor | Photorespiratory | -1.6  0.01 | -1.5  0.03 |
| No.13 | 39.6/5.59 | 46.2/5.46 | 26.40%(8) | BE497239 | Delta-aminolevulinic acid dehydratase | Chloroplast development, regulation of chlorophyll | -1.7  0.00 | -1.9  0.05 |
| No.03 | 61.9/5.90 | 64.1/6.35 | 30.28%(8) | CJ870803 | Pentatricopeptide (PPR) repeat-containing protein | Organelle biogenesis and plant development. | -1.8  0.00 | -1.8  0.00 |
| No.47 | 51.6/5.80 | 47.0/5.90 | 30.75%(9) | CJ787790 | Expressed protein | Unknown | -  0.00 | -  0.00 |
| No.20 | 48.4/5.70 | 41.4/6.03 | 20.75%(5) | CV778959 | Expressed protein | Unknown | -2.1  0.02 | -2.2  0.01 |

^a^Experimental relative molecular mass (KDa) /isoelectric point.

^b^Theoretical relative molecular mass (KDa) /isoelectric point.

^c^Sequence coverage by peptide mass ﬁngerprinting using MALDI-TOF MS. PM, number of peptides matched.

^d^Protein fold change compared with the control. _ represents no significant change of spot abundance between mock and *F. graminearum* in the corresponding material; / represents no spot has been detected in the corresponding material.  represents the proteins that show qualitative change after the infection.

^e^A *t*-test was performed to determine the *p*-value.
