# Supplementary material for: Resistance to Hemi-Biotrophic F. graminearum Infection Is Associated with Coordinated and Ordered Expression of Diverse Defense Signaling Pathways
Source: PLoS One. 2011 Apr 20;6(4):e19008. doi: 10.1371/journal.pone.0019008 (PMC3080397; doi:10.1371/journal.pone.0019008)
Supplement: Table S2 — Differentially expressed genes after F. graminearum infection identified by in silico Northerns and their functional annotations. (DOC) [file pone.0019008.s003.doc]

**Table S2.** Differentially expressed genes after *F. graminearum* infection identified by *in silico* Northerns and their functional annotations

|  | | | |
| --- | --- | --- | --- |
| **ID number** | **GenBank accession no.** | **Annotation** | **Putative function description** |
| **Resistance or defense related** | | | |
| ***Genes associated with SA biosynthesis or SA signaling*** | | | |
| IGS001* | 45798589 | PR2-like protein, SA inducible |  |
| IGS002* | 45797950 | R2R3 Myb-like protein | Hypersensitive and PCD regulation in response to pathogen attack through inducing SA biosynthesis (Vailleau et al. 2002). |
| IGS003 | 17147399 | PR2 | Defense related |
| IGS004 | 17142168 | PR2-like | Defense related |
| IGS005 | 9839201 | PR1 like | Defense related |
| IGS006 | 45801581 | Thaumatin-like protein | Defense related (Reiss and Horstmann, 2001). |
| IGS007 | 45799497 | Thaumatin-like protein | Defense related (Reiss and Horstmann, 2001). |
| IGS008 | 45800761 | Thaumatin-like protein | Defense related, has an antifungal activity (Kuwabara et al. 2002). |
| ***Genes associated with JA/ET biosynthesis or JA/ET signaling*** | | | |
| IGS009* | 45798338 | JASMONATE INSENSITIVE 1-like protein | Required for JA regulated defense signaling (Boter et al. 2004). |
| IGS010* | 17144184 | Short-chain alcohol dehydrogenase | Essential for ABA biosynthesis. ABA is an essential signal for plant resistance to pathogens affecting JA biosynthesis (Adie et al. 2007). |
| IGS011 | 45802024 | Pathogenesis-related protein 4 | Disease resistance related (Fiocchetti et al. 2008). |
| IGS012 | 17143230 | PR1.2 | Defense related, induced in a SA independent manner (Molina et al. 1999). |
| IGS013 | 17146964 | PR1.1 | Defense related, induced in a SA independent manner (Molina et al. 1999). |
| IGS014 | 45801856 | Chitinase I like | FHB defense response, ET inducible (Wang et al. 2009). |
| IGS015 | 9838571 | Chitinase IV like | PR3 |
| IGS016 | 9838707 | AtPFT1-like transcription coactivator | Key regulator of JA-dependent defense (Kidd et al. 2009). |
| IGS017 | 45800594 | ERF1-like | Positive regulator of disease resistance responses in the JA/ET pathways (Liang et al. 2007; Chen et al. 2008). |
| IGS018 | 9839024 | Iron/ascorbate-dependent oxidoreductase | Might be involved in ethylene biosynthesis (Bogacki et al. 2008). |
| ***Genes related to PA biosynthesis or PA signaling*** | | | |
| IGS019* | 9838994 | Diacylglycerol kinase 1 | Phospholipids signal transduction. SA inducible. |
| IGS020* | 22300167 | Phospholipase D | Phospholipids signal transduction. SA inducible. |
| IGS021 | 17147119 | Phospholipid synthesis protein like | Involved in PA biosynthesis and signaling (Mita et al. 2006). |
| ***Genes related to cross-talk of signaling pathways*** | | | |
| IGS022* | 15773280 | Putative BRI1-associated receptor kinase | Integration of diverse perception events into downstream PAMP responses leading to systemic immunity (Kemmerling et al. 2007). |
| IGS023 | 9839033 | Putative BRI1-associated receptor kinase like | Integration of diverse perception events into downstream PAMP responses leading to systemic immunity (Kemmerling et al. 2007). |
| IGS024 | 22304326 | OsMKP1-like | Negative regulators of MAPK cascade, regulated by CaM to repress ICS1 mediated SA biosynthesis (Katou et al. 2007; Bartels et al. 2009). |
| ***Genes related to antimicrobial compound synthesis or detoxification*** | | | |
| IGS025* | 45800035 | Cytochrome P450 CYP72A16 | Probably related to antimicrobial compound synthesis. |
| IGS026* | 45802908 | Cytochrome P450 CYP72A1 | Probably related to antimicrobial compound synthesis. |
| IGS027* | 22301296 | Farnesol synthase | Related to sesquiterpene phytoalexin biosynthesis. |

**Table S2.** (continued)

|  | | | |
| --- | --- | --- | --- |
| **ID number** | **GenBank accession no.** | **Annotation** | **Putative function description** |
| IGS028* | 92235533 | Cytochrome P450 99A2 | Production of phytoalexin momilactones |
| IGS029* | 9838737 | Cytochrome P450 99A3 | Production of phytoalexin momilactones |
| IGS030* | 92234638 | Cytochrome P450 99A1 | Production of phytoalexin momilactones |
| IGS031* | 45800675 | Cytochrome P450 71D8 | Production of phytoalexin camalexin |
| IGS032* | 15772673 | Elicitor inducible cytochrome P450 | Production of phytoalexin momilactones |
| IGS033* | 17146292 | OsKSL4-like protein | Probably involved in phytoalexin biosynthesis |
| IGS034* | 20298831 | OsCPS2 like protein | Involved in phytoalexin biosynthesis (Prisic et al. 2004). |
| IGS035* | 92236030 | Benzoxazineless | Biosynthesis of antimicrobial compound DIBOA (Frey et al. 1997). |
| IGS036* | 22303890 | Tyrosine/dopa decarboxylase 1 | Production of secondary metabolite amides, a component of cell wall (Facchini et al. 1999). |
| IGS037* | 45802845 | Tyrosine/dopa decarboxylase 2 | Production of secondary metabolite amides, a component of cell wall (Facchini et al. 1999). |
| IGS038* | 92234695 | Obtusifoliol 14alpha-demethylase | Production of antimicrobial compounds (Qi et al, 2006). |
| IGS039* | 45799276 | UDP-glucoronosyl and UDP-glucosyl transferase | Detoxification of Fusarium mycotoxin DON (Poppenberger et al. 2003). |
| IGS040* | 92234679 | MATE efflux family protein | Detoxification of secondary metabolites and defense-related (Nawrath et al. 2002). |
| IGS041 | 45800796 | MATE efflux family protein | Detoxification of secondary metabolites and defense-related (Nawrath et al. 2002). |
| IGS042 | 17145669 | Cytochrome P450 99A2 | Production of phytoalexin momilactones |
| IGS043 | 9838564 | Cytochrome P450 CYP51H10 | Production of antimicrobial compound avenacins (Qi et al. 2006). |
| IGS044 | 9838859 | Cytochrome P450 CYP51H11 | Production of antimicrobial compound avenacins (Qi et al. 2006). |
| IGS045 | 45802200 | Benzoxazineless 2 like | Biosynthesis of antimicrobial compound DIBOA (Frey et al. 1997). |
| IGS046 | 45800565 | Cytochrome P450 72A124 | Indole and ipecac alkaloid biosynthesis (Ueno et al. 2004). |
| IGS047 | 17145531 | AtOPR1 homolog | Detoxification, induced by JA (Sobajima et al. 2002). |
| IGS048 | 9838636 | AtOPR2 homolog | Detoxification, induced by JA (Sobajima et al. 2002). |
| IGS049 | 9838612 | OsKSL4 like | Production of the dual function phytoalexin/allelochemical momilactones, induced by MeJA. |
| IGS050 | 9839094 | UDP-glucosyltransferase | Detoxification of Fusarium mycotoxin DON (Poppenberger et al. 2003). |
| IGS051 | 45799907 | UDP-glucosyltransferase like | Detoxification of Fusarium mycotoxin DON (Poppenberger et al. 2003). |
| IGS052 | 17141957 | Glucosyltransferase-10 like | Detoxification of Fusarium mycotoxin DON (Desmond et al. 2008). |
| IGS053 | 17147064 | Glyoxalase I family protein | Detoxification of methylglyoxal, stress related and fusarium inducible (Lin et al. 2009). |
| IGS054 | 17147281 | Putative tryptophan synthase beta chain | Tryptophan synthesis. Coregulated with the production of phytoalexin camalexin (Zhao and Last 1996). |
| IGS055 | 45801908 | Putative ferredoxin-3 | Anti-microbial protein (van den Berget al. 2007; Liau et al. 2003). |
| IGS056 | 17145989 | Putative UMP synthase | Pyrimidine biosynthesis, important for cell wall polysaccharide metabolism or toxic compound biosynthesis in plants (Santoso and Thornburg, 1998). |
| IGS057 | 45799173 | Putative chalcone synthase | Flavonoid biosynthesis, SA/MeJA inducible (Salzman et al. 2005). |
| IGS058 | 45801875 | Leucoanthocyanidin dioxygenase | Antioxidant flavonoids anthocyanin biosynthesis, SA+MeJA inducible (Salzman et al. 2005). |
| IGS059 | 45802756 | Putative flavonol 4-sulfotransferase | JA inducible |

**Table S2.** (continued)

|  | | | |
| --- | --- | --- | --- |
| **ID number** | **GenBank accession no.** | **Annotation** | **Putative function description** |
| ***Genes related to antioxidative stress*** | | | |
| IGS060* | 15773259 | ATOSA1-like | Oxidative stress-related (Jasinski et al. 2008). |
| IGS061* | 22302067 | NADPH: quinone oxidoreductase | Defense against oxidative stress (Babiychuk et al. 1995). |
| IGS062 | 45803221 | Glutathione S-transferase, tau class | Pathogen infection induced (Wagner et al. 2002). |
| IGS063 | 9838561 | Glutathione S-transferase 1 homolog, phi class | Pathogen infection induced (Wagner et al. 2002). |
| IGS064 | 9838974 | Glutathione S-transferase F4 homolog, phi class | Pathogen infection induced (Wagner et al. 2002). |
| IGS065 | 9838681 | Glutathione S-transferase, tau class | Pathogen infection induced (Wagner et al. 2002). |
| IGS066 | 45802975 | Glutathione S-transferase GSTU6-like, tau class | Antioxidative stress? JA inducible |
| IGS067 | 9838635 | Class III peroxidase 30 | ROS production to reinforce the cell wall and or by direct toxicity to the fungus (Liu et al. 2005). |
| IGS068 | 45800398 | Class III peroxidase 112 | ROS production to reinforce the cell wall and or by direct toxicity to the fungus (Simonetti et al. 2009; Liu et al. 2005). |
| IGS069 | 45800834 | Class III peroxidase 22-like | Defense against pathogens (Hiraga et al. 2001). |
| IGS070 | 9838771 | Class III peroxidase 107-like | Defense against pathogens (Hiraga et al. 2001). |
| IGS071 | 9838509 | Monodehydroascorbate reeducates-like | Oxidative stress response, SA inducible (Yoon et al. 2004). |
| IGS072 | 45799587 | Blue copper-binding protein homolog | Oxidative stress defense, induced by stripe rust and powdery mildew (Coram et al. 2009; Gjetting et al. 2007) |
| IGS073 | 17144461 | Alternative oxidase AOX1b-like | PCD controlling under oxidative stress, regulated by SA, JA, ET and ROS through a complex network (Hanqing et al. 2010). |
| IGS074 | 45800026 | Peptide methionine sulfoxide reductase-like | Protect cell from oxidative damage |
| ***Resistance gene analogs or kinase proteins*** | | | |
| IGS075* | 9838709 | Similar to stripe rust resistance protein Yr10 | Disease resistance related |
| IGS076* | 17145040 | Rust resistance gene ABC1041 | Disease resistance related |
| IGS077* | 17921443 | Mla-like protein | Disease resistance related |
| IGS078* | 17142994 | Hv1LRR2 | Disease resistance related |
| IGS079* | 45799531 | LRR14 | Disease resistance related |
| IGS080* | 22303183 | NB-ARC domain-containing protein | Disease resistance related |
| IGS081* | 17143314 | NB-ARC domain containing protein | Disease resistance related |
| IGS082* | 22303910 | NB-ARC domain containing protein | Disease resistance related |
| IGS083* | 45803172 | Lr1 disease resistance protein like | Disease resistance-related |
| IGS084* | 22304514 | NBS-LRR disease resistance protein homologue | Disease resistance-related |
| IGS085* | 45800100 | Cf-2.3 like | Disease resistance-related |
| IGS086* | 45803249 | Putative disease resistance gene homolog 9N | Disease resistance-related |
| IGS087* | 17143134 | I2-like | Disease resistance-related |
| IGS088* | 45801213 | Disease resistance protein I2 | Disease resistance-related |
| IGS089* | 92235176 | TIR/P-loop/LRR disease resistance protein-like protein | Disease resistance-related |
| IGS090* | 45799827 | Protein kinase domain containing protein | Probably resistance related |

**Table S2.** (continued)

|  | | | |
| --- | --- | --- | --- |
| **ID number** | **GenBank accession no.** | **Annotation** | **Putative function description** |
| IGS091* | 22304538 | Leucine rich repeat containing protein kinase | Probably resistance related |
| IGS092* | 92235908 | Leucine Rich Repeat family protein | Probably resistance related |
| IGS093* | 17921391 | Receptor kinase Lecrk | Disease resistance-related (Chen et al. 2006). |
| IGS094* | 92236341 | Receptor kinase Lecrk | Disease resistance-related (Chen et al. 2006). |
| IGS095* | 22302655 | Receptor kinase Lecrk | Disease resistance-related (Chen et al. 2006). |
| IGS096* | 45800464 | Nucleoid DNA-binding protein cnd41 | Defense related transcription factor (Mysore et al. 2002). |
| IGS097* | 17145809 | Receptor-like protein kinase 1 | Signal transduction, might be involved in defense. |
| IGS098* | 45802324 | S-receptor like kinase | Probably involved in signal transduction in defense (Pastuglia et al. 1997). |
| IGS099* | 45799363 | Receptor-like protein kinase 4 | Probably involved in signal transduction in defense |
| IGS100* | 45803153 | Serine/threonine kinase protein | Probably involved in signal transduction in defense |
| IGS101* | 45799513 | Serine/threonine protein phosphatase 2A -like protein | Probably involved in signal transduction in defense |
| IGS102 | 45798955 | Receptor kinase RK20-like | Involved in plant–microbe interactions (Chen et al. 2004). |
| IGS103 | 45799513 | Serine/threonine protein phosphatase 2A -like protein | Probably involved in signal transduction in defense |
| ***Genes related Ca signaling*** | | | |
| IGS104* | 17143775 | Calcium-transporting ATPase 13, plasma membrane-type | Ca^2+^ transporter, involved in signaling pathways of the hypersensitive response (Nemchinov et al. 2008). |
| IGS105* | 22303322 | C2 domain-containing protein | Ca^2+^-dependent membrane-targeting module, defense related (Kim et al. 2008). |
| IGS106* | 45800261 | MDR-like ABC transporter | Induced by breaking of calcium homeostasis (Sasaki et al. 2002). |
| IGS107 | 17141871 | Annexin P35-like | Play a link role between Ca^2+^ signaling and diverse membrane-related functions (Vandeputte et al. 2007). |
| ***Genes related to post-transcriptional regulation and associated with defense*** | | | |
| IGS108* | 22302964 | RNA-dependent RNA polymerase | SA and JA inducible, functioning downstream of NPR1 (Quilis et al. 2008). |
| IGS109* | 17144407 | Mitochondrial transcription termination factor | Related to RNA Adenine Methyltransferases and binds S-Adenosylmethionine, and is statistically associated with quantitative resistance (Wisser et al. 2005). |
| IGS110* | 45801246 | Mitochondrial transcription termination factor | Related to RNA Adenine Methyltransferases and binds S-Adenosylmethionine, and is statistically associated with quantitative resistance (Wisser et al. 2005). |
| IGS111* | 45802465 | Mitochondrial transcription termination factor | Related to RNA Adenine Methyltransferases and binds S-Adenosylmethionine, and is statistically associated with quantitative resistance (Wisser et al. 2005). |
| IGS112* | 17145385 | Argonaute-like protein | Involved in gene-silencing pathways. |
| IGS113 | 45802880 | RNA methylase-like protein | Post-transcriptional regulation, might involve in plant defense (Katiyar-Agarwal, 2010). |
| ***Genes for cell wall fortification*** | | | |
| IGS114* | 45801813 | Aspartate-semialdehyde dehydrogenase | Synthesis of cell wall component |
| IGS115 | 17146379 | Cinnamoyl-CoA reductase | Lignin biosynthesis, defense response and regulated by small GTPase Rac (Kawasaki et al. 2005). |
| IGS116 | 9839171 | Caffeic acid-3-O-methyltransferase | Lignin biosynthesis |
| IGS117 | 17144819 | Putative ferulate 5-hydroxylase | Guaiacyl-syringyl lignin accumulation (Meyer et al. 1996). |
| ***Other defense related genes*** | | | |
| IGS118* | 22302321 | Putative hypersensitivity-related protein | Defense related, probably SA inducible. |
| IGS119* | 22302326 | Putative cell death associated protein | Defense related |
| IGS120* | 20298824 | Serine protease inhibitor | Inducted by beta-amino-butyric acid and pathogen infection, probably JA inducible. |

**Table S2.** (continued)

|  | | | |
| --- | --- | --- | --- |
| **ID number** | **GenBank accession no.** | **Annotation** | **Putative function description** |
| IGS121* | 92234969 | Epoxide hydrolase | Defense related (Gom et al. 2004). MeJA inducible (Gomi et al. 2003). |
| IGS122* | 17143479 | Ribosomal RNA apurinic site specific lyase 1 | Ribosome-inactivation, defense against pathogens (Corrado et al. 2005). |
| IGS123* | 45799162 | Ribosomal RNA apurinic site specific lyase 2 | Ribosome-inactivation, defense against pathogens (Corrado et al. 2005). |
| IGS124* | 92236838 | WIR1A | Integrate membrane protein. Pathogen-inducible. |
| IGS125* | 20299202 | Integral membrane protein | Signal receptors or ion transporters. Some R genes are integral membrane protein. |
| IGS126* | 92235707 | HAK4 like | Potassium transporter, Fusarium inducible (Kruger et al. 2002; Bernardo et al. 2007). |
| IGS127* | 15773364 | Membrane protein | Some membrane proteins are related to defense |
| IGS128* | 22302509 | B-keto acyl reductase | Induced systemic resistance related (Verhagen et al. 2004). |
| IGS129* | 45798845 | AMP-binding enzyme | Defense-related (Zhang et al. 2009). |
| IGS130* | 22302315 | Paramyosin-like protein | Paramyosin was identified as the major immunogen. |
| IGS131* | 22301211 | Dolichyl-phosphate-mannose-glycolipid alpha-mannosyltransferase | N-glycan biosynthesis (Pattison and Amtmann 2008). |
| IGS132* | 45801657 | Lipase | Defense related (Shah, 2005). |
| IGS133* | 45799860 | Very-long-chain fatty acid condensing enzyme CUT1 | Defense related (Raffaele et al. 2009). |
| IGS134* | 31157924 | Microtubule associated protein family protein | Restricting tobamoviral infections in Arabidopsis |
| IGS135* | 45801145 | LysM domain containing protein | Bacterial cell wall degradation (Buist et al. 2008). |
| IGS136* | 45803055 | Phosphoenolpyruvate carboxylase | Related to phorsphorylation/dephosphorylation associated signaling in PVY (NTN) infected tobacco (Müller et al. 2009). |
| IGS137 | 17144690 | Putative ornithine decarboxylase | Polyamine biosynthesis, SA independent defense reaction (Yoo et al. 2004). |
| IGS138 | 17145728 | Wali1 | Metallothionein, hypersensitive reaction related (Butt et al. 2001). |
| IGS139 | 45801008 | NAC domain transcription factor ATNAC2-like | Regulated by ET, auxin and JA, stress related (Seo et al. 2009; Oh et al. 2005; He et al. 2005) |
| IGS140 | 45798523 | (1,3; 1,4) beta glucanase | Defense related, MeJA inducible (Akiyama et al. 2009). Response to infection with *B. cinerea* (Renault et al 2000). |
| IGS141 | 45800551 | PR17c-like | PR17 family includes NtPRp27, which is more sensitive to JA and ET. |
| IGS142 | 20298686 | PDR-type ABC transporter | Responsive to SA and MeJA (Eichhorn et al. 2006), might confer *F. graminearum* resistance in wheat (Kobae et al. 2006; Yi et al. 2009). |
| IGS143 | 9838742 | Putative selenium binding protein OsSBP-like | JA and SA inducible, involved in pathogen resistance (Dutilleul et al. 2008; Sawada et al. 2002, 2004). |
| IGS144 | 20299223 | RING-H2 zinc finger family protein | Developmental processes and plant defense responses (Liu et al. 2008). |
| IGS145 | 17142980 | Alpha-1, 6-mannosyl-glycoprotein 2-beta-N-acetylglucosaminyltransferase | N-glycan biosynthesis |
| IGS146 | 17145605 | Putative protease inhibitor | Proteinase inhibitor, disease resistance related (Steiner-Lange et al. 2003). |
| IGS147 | 45802993 | MPI homolog | Proteinase inhibitor, defense response, JA inducible (Cordero et al. 1994). |
| IGS148 | 17146666 | Wali6 | Bowman-Birk proteinase inhibitor, SA+MeJA inducible (Salzman et al. 2005). |
| IGS149 | 45803029 | Wali3-like | Putative wound-induced protease inhibitor |
| IGS150 | 17146187 | Dirigent-like protein |  |
| IGS151 | 9838550 | Putative 3-beta hydroxysteroid dehydrogenase/isomerase | Steroid biosynthesis, Fusarium inducible (Bernardo et al. 2007). |

**Table S2.** (continued)

|  | | | |
| --- | --- | --- | --- |
| **ID number** | **GenBank accession no.** | **Annotation** | **Putative function description** |
| IGS152 | 45799700 | Type 2 non-specific lipid transfer protein | Might involve in defense against pathogens (Salcedo et al. 2007). |
| IGS153 | 17142090 | B12D protein | Basal defense-related (Caldo et al. 2006). |
| IGS154 | 9838511 | AtSTP4-like | Monosaccharide transporter, involved in response to powdery mildew infection (Truernit et al. 1996; Fotopoulos et al. 2003) |
| IGS155 | 22300527 | Maltose excess protein 1 | Essential for the conversion of starch to sucrose. Sucrose accumulation is related to defense response (Gómez-Ariza et al. 2007). |
| IGS156 | 17146625 | Sugar transporter protein like | Defense related (Nørholm et al. 2006). |
| IGS157 | 9838674 | Plant viral-response family protein-like | Defense response |
| IGS158 | 45800650 | MTN3 homolog | Disease resistance related (Chu et al. 2005, 2006). |
| IGS159 | 45802163 | Papain-like cysteine proteinase C1A subfamily protein | Might involve in fungal resistance (Tian et al. 2007; Shabab et al. 2008). |
| IGS160 | 45800172 | OsHAK5-like | Potassium transporter, Fusarium inducible (Kruger et al. 2002; Bernardo et al. 2007). |
| IGS161 | 9838690 | OsBi1-like | Brown planthopper-induced resistance protein, insects resistance related (Wang et al. 2004). |
| IGS162 | 17146148 | Putative CPRD2 | Pathogen inducible (González-Candelas et al. 2009). |
| IGS163 | 17143363 | Endo-1, 4-beta glucanase like | Cell wall modification, necessary for lignification (Tsabary et al. 2003). |
| **Genes not known for association with resistance or defense** | | | |
| IGS164* | 45803069 | SAUR52 - auxin-responsive SAUR family member | Auxin response related |
| IGS165* | 45803192 | Secretory carrier-associated membrane protein 4 | Functioning in membrane trafficking, biotic stress tolerance related. |
| IGS166* | 22301529 | MYB-like transcription factor | Stress response regulator |
| IGS167* | 9838655 | Fatty acyl coA reductase | Wax biosynthesis (Rowland et al. 2006). |
| IGS168* | 45802604 | Phosphoserine phosphatase | Stress response? Catalyzes the last step in the biosynthesis of serine from carbohydrates. |
| IGS169* | 45800477 | tRNA-splicing endonuclease positive effector | tRNA processing |
| IGS170* | 20298829 | Splicing factor 3B subunit 2 like | RNA splicing |
| IGS171* | 45798962 | Succinate dehydrogenase complex, subunit A | Key protein of the mitochondria electron transfer chain |
| IGS172* | 22304561 | Sucrose: fructan 6-fructosyltransferase | Key enzyme for diverting carbon from sucrose to fructan |
| IGS173* | 22304104 | Alpha-glucan water dikinase 1, chloroplastic | Freezing tolerance / starch degradation |
| IGS174* | 45798128 | Vegetative storage protein win4.5 | Storage protein (Davis et al. 1993). |
| IGS175* | 45800750 | Vacuolar protein sorting 13C protein | Protein destination and storage |
| IGS176* | 22302280 | Aminoacyl peptidase | Proteinase, protein destination and storage. |
| IGS177* | 9838845 | F-box domain containing protein 1 | Plant growth/development and responses to environmental stimuli. |
| IGS178* | 45797971 | F-box domain containing protein 2 | Plant growth/development and responses to environmental stimuli. |
| IGS179* | 45799479 | Cysteinyl-tRNA synthetase | Synthesis of cysteinyl-transfer RNA |
| IGS180* | 9839115 | Histidyl-tRNA synthetase | Synthesis of histidyl-transfer RNA |
| IGS181* | 17143202 | GA2-oxidase | GA biosynthesis |
| IGS182* | 15772826 | Putative pentatricopeptide repeat containing protein |  |
| IGS183* | 92236078 | Hypothetical protein |  |
| IGS184* | 92236647 | Hypothetical protein |  |
| IGS185* | 92236332 | Pollen allergen-like |  |

**Table S2. (continued)**

|  | | | |
| --- | --- | --- | --- |
| **ID number** | **GenBank accession no.** | **Annotation** | **Putative function description** |
| IGS186* | 9839182 | C2H2-type protein | Signaling transduction-related |
| IGS187* | 45798593 | PAS domain containing protein | Sensing external signals |
| IGS188* | 17146251 | HVA22 family protein | Stress response protein |
| IGS189* | 92234938 | Unclassified retrotransposon protein 1 | Transposition |
| IGS190* | 45800988 | Unclassified retrotransposon protein 2 | Transposition |
| IGS191* | 22303095 | En/Spm sub-class transposon protein | Transposition |
| IGS192* | 17144855 | Putative transposon protein | Transposition |
| IGS193* | 15772903 | Putative LINE subclass retrotransposon protein | Transposition |
| IGS194* | 17145887 | Polyprotein | Transposition |
| IGS195* | 45798982 | Reverse transcriptase | Transposition |
| IGS196* | 45800800 | Putative Ty1-copia subclass retrotransposon protein | Transposition |
| IGS197 | 9839035 | Gibberellin 20-oxidase like |  |
| IGS198 | 45800968 | Auxin-repressed protein ARP1-like | Might be regulated by auxin positively or negatively (Kim et al. 2007). |
| IGS199 | 45798190 | Nucleotide pyrophosphatase/phosphodiesterase | Responses to phosphate-deprived environment (Zhu et al. 2005; Hur et al. 2009). |
| IGS200 | 45800548 | WRKY27 transcription factor. | Negatively influences symptom development of a vascular pathogen |
| IGS201 | 22302828 | Multiple inositol polyphosphate phosphatase PhyIIa2 homolog | Inositol phosphate metabolism |
| IGS202 | 20298693 | Putative T-complex protein 11 | Protein folding and assembly |
| IGS203 | 17143310 | 2-oxoisovalerate dehydrogenase alpha subunit | Conversion of alpha-keto acids to acyl-CoA and CO_2_ |
| IGS204 | 9838986 | Protein tyrosine phosphatase-like protein PASTICCINO 2B-like | Plant development |
| IGS205 | 17144928 | ZIP zinc/iron transport family protein | Zinc homeostasis |
| IGS206 | 45800841 | NADPH-protochlorophyllide oxidoreductase B-like | Maintaining Chl levels throughout angiosperm development (Armstrong et al. 1995). |
| IGS207 | 17151510 | Putative nitrate transporter NRT1 | Transporter, nitrate homeostasis |
| IGS208 | 45800527 | Pyruvate dehydrogenase kinase 1-like | Negative regulator of the mitochondrial pyruvate dehydrogenase (PDH) complex cycle. |
| IGS209 | 17145393 | Putative metal ion binding protein | Unknown |
| IGS210 | 17147138 | Putative oxidase-like | Unknown |
| IGS211 | 45798555 | FAD binding domain containing protein | Unknown |
| IGS212 | 17144378 | Hydrolase, alpha/beta fold family protein | Required for energy stress activation of the sigma (B) transcription factor (Brody et al. 2001). |
| IGS213 | 9838766 | 91A protein-like | Unknown |
| IGS214 | 45801827 | Thaumatin-like cytokinin-binding protein | Unknown |
| IGS215 | 45802918 | GATA transcription factor |  |
| IGS216 | 45802496 | Putative drug transporter |  |
| IGS217 | 45798979 | Putative oligopeptide transporter PTR2-B like | Unknown |
| IGS218 | 22301747 | Putative HAD-superfamily hydrolase subfamily IA, variant 3 | Unknown |
| IGS219 | 45798877 | MAPEG family protein |  |

*Induced genes responding to *F. graminearum* infection.
