# Supplementary material for: Resistance to Hemi-Biotrophic F. graminearum Infection Is Associated with Coordinated and Ordered Expression of Diverse Defense Signaling Pathways
Source: PLoS One. 2011 Apr 20;6(4):e19008. doi: 10.1371/journal.pone.0019008 (PMC3080397; doi:10.1371/journal.pone.0019008)
Supplement: Table S3 — Expression correlations of the genes examined in Wangshuibai and its susceptible mutant Meh0106 involved in the early signaling events after F. graminearum infection. (DOC) [file pone.0019008.s004.doc]

**Table S3.** Expression correlations of the genes examined in Wangshuibai and its susceptible mutant Meh0106 involved in the early signaling events after *F. graminearum* infection.

W: Wangshuibai, m: Meh0106.

* Correlation is significant at p= 0.05 (2-tailed).

** Correlation is significant at p= 0.01 (2-tailed).

|  | 12-OPR3 | ACCO | CaM | NADPH oxidase | DAGK | PAO | PLD | CCOMT | CHS |
| --- | --- | --- | --- | --- | --- | --- | --- | --- | --- |
| PAL | 0.174(W)  0.785(m) | 0.672(W)  0.003(m) | 0.910(*)(W)  0.127(m) | 0.874(*)(W)  -0.325(m) | 0.921(**)(W)  0.523(m) | 0.274(W)  -0.187(m) | 0.411(W)  -0.397(m) | 0.161(W)  0.753(m) | 0.335(W)  0.856(*)(m) |
| 12-OPR3 |  | 0.173(W)  0.273(m) | -0.012(W)  0.312(m) | 0.094(W)  -0.182(m) | 0.280(W)  0.563(m) | 0.741(W)  0.108(m) | 0.722(W)  -0.124(m) | 0.826(*)(W)  0.904(*)(m) | 0.957(**)(W)  0.744(m) |
| ACCO |  |  | 0.394(W)  0.728(m) | 0.251(W)  0.409(m) | 0.413(W)  0.668(m) | 0.256(W)  0.744(m) | 0.740(W)  0.355(m) | 0.361(W)  0.001(m) | 0.267(W)  0.040(m) |
| CaM |  |  |  | 0.966(**)(W)  0.085(m) | 0.956(**)(W)  0.882(*)(m) | 0.025(W)  0.844(*)(m) | 0.082(W)  0.626(m) | -0.186(W)  0.152(m) | 0.174(W)  0.471(m) |
| NADPH oxidase |  |  |  |  | 0.961(**)(W)  0.139(m) | 0.072(W)  0.574(m) | 0.025(W)  0.565(m) | -0.074(W)  -0.027(m) | 0.234(W)  -0.471(m) |
| DAGK |  |  |  |  |  | 0.231(W)  0.710(m) | 0.273(W)  0.432(m) | 0.066(W)  0.460(m) | 0.440(W)  0.692(m) |
| PAO |  |  |  |  |  |  | 0.755(W)  0.881(*)(m) | 0.693(W)  0.080(m) | 0.819(*)(W)  0.077(m) |
| PLD |  |  |  |  |  |  |  | 0.747(W)  0.018(m) | 0.779(W)  -0.059(m) |
| CCOMT |  |  |  |  |  |  |  |  | 0.709(W)  0.707(m) |
