# Supplementary material for: Resistance to Hemi-Biotrophic F. graminearum Infection Is Associated with Coordinated and Ordered Expression of Diverse Defense Signaling Pathways
Source: PLoS One. 2011 Apr 20;6(4):e19008. doi: 10.1371/journal.pone.0019008 (PMC3080397; doi:10.1371/journal.pone.0019008)
Supplement: Table S4 — Primer sequences used in qRT-PCR. (DOC) [file pone.0019008.s005.doc]

**Table S4.** Primer sequences used in qRT-PCR.

|  | | |
| --- | --- | --- |
| **Name** | **Forward primer** | **Reverse primer** |
| *12-OPR3* | 5'-CCATAAACGCCATCAAAGCAGG-3' | 5'-TGCATCGGGTTCGAGTCATAGG-3' |
| *ACO* | 5'-AGCAGGATCTTCGTCAACATCG-3' | 5'-ATCGTGCCGAATCCGTGAAC-3' |
| *BIK1* | 5'-TCAGTCTTCAAGGGGTGGATCG-3' | 5'-GGGCAATTTTCATCCGAAGG-3' |
| *Ca^2+^ATPase* | 5'-TCCTGATGAAAAGGGCAACC-3' | 5'-GCACAGCACCAGATACAAATGC-3' |
| *CaM* | 5'-TCAGCCTCTTCGACAAGGATG-3' | 5'-CGAGATGAAGCCGTTCTGGT-3' |
| *CCOMT* | 5'- ATCCTCGACAAGCTCATCGC-3' | 5'- CCTGATGGAGTCCCGTTTCTT-3' |
| *Chitinase 1* | 5'-GGGCGGTCCAACTACGATCTT-3' | 5'-CGTAACAAGTGCATACACTGTGATGG-3' |
| *CHS* | 5'-CGAATGGAACAACCTCTTCTGG-3' | 5'-GTCCAAATCCCATCATCACCC-3' |
| *CYP71D8* | 5'-CCCTTCACCAATCGACCTCCAAG-3' | 5'-TAGAGGAGCGGGACTATGGCTAGG-3' |
| *DGK* | 5'-GGCACGAGGAAGAAACAAGACA-3' | 5'-CAGGTAACTTGAAGGTATCCGCA-3' |
| *EDS1* | 5'-TGAGATGTTGCGAAGGCGTG-3' | 5'-CGAGTTTACCCACCCTGTCTTTC-3' |
| *ERF1* | 5'-CACCTTGACCTCCTCCTCTTCGC -3' | 5'-TTGTTCCCTTTGGACGCCAGG -3' |
| *Glu2* | 5'-CCAACATCTACCCGTACCTGGC-3' | 5'-GACACCACGAGCTTCACGTTG-3' |
| *ICS1* | 5'-AGAAATGAGGACGACGAGTTTGAC-3' | 5'-CCAAGTAGTGCTGATCTAATCCCAA-3' |
| *MKP-1* | 5'-GGGCACCATCTATTCACAAAGC-3' | 5'-CACTTCTGCTGGCATTAGGAGC-3 |
| *MPK4* | 5'-CGTTTGACAACCACATCGACG-3' | 5'-TTGGCTTCAGATCACGGTGC-3' |
| *MYC2* | 5'-AGCAGGTGGCGGTGAAGAT-3' | 5'-CATCCATCCATCCATCCTCC-3' |
| *NADPH oxidase* | 5'-ATGCTCCAGTCCCTCAACCAT-3' | 5'-TTCTCCTTGTGGAACTCGAATTT-3' |
| *NPR1* | 5'-CTGTCCGACTTTGTGAGCATAGC-3' | 5'-CCCGCTGTCATTCTTCAGGTTG-3' |
| *PAL* | 5'-TTGATGAAGCCGAAGCAGGACC-3' | 5'-ATGGGGGTGCCTTGGAAGTTGC-3' |
| *PAO* | 5'-TGGTGGTCCAACCGATTCTTC-3' | 5'-TGCTCGTTCATTTAGCCTCAGC-3' |
| *PLD* | 5'-TGGGCTCGGCAAACATCAATC-3' | 5'-TCGTCAGCGAATCAGGAAGCG-3' |
| *PR3* | 5'-CCTCCATTATCTCGCAGTCGCTC-3' | 5'-CGCCGTAGTTGTAGAACCCCTTG-3' |
| *SAMDC* | 5'-'ACTGGGCTGGACAAGAAGAAGG | 5'-TTGTTGCAGTCGTATGCCTCG-3' |
| *WRKY33* | 5'-TGACAACGAGGGCAGTTCAGG-3' | 5'-CTTGTAGTAGCTCCTGGGGTTGG-3' |
